# Early Postnatal Infection With *Human Cytomegalovirus* Has Long‐Term Consequences on Brain Structure of Former Preterm Born Children

**DOI:** 10.1002/brb3.70985

**Published:** 2025-10-15

**Authors:** Meike Müller, Karen Lidzba, Christian Gaser, Till‐Karsten Hauser, Rangmar Goelz, Klaus Hamprecht, Marko Wilke

**Affiliations:** ^1^ Department of Neuropediatrics, General Pediatrics, Diabetology, Endocrinology, Social Paediatrics University Children's Hospital Tübingen Tübingen Germany; ^2^ Experimental Pediatric Neuroimaging, University Children's Hospital Tübingen and Department of Neuroradiology University Hospital Tübingen Tübingen Germany; ^3^ Division of Neuropaediatrics, Development and Rehabilitation, Department of Paediatrics, Inselspital University Hospital, University of Bern Bern Switzerland; ^4^ Department of Psychiatry and Psychotherapy Jena University Hospital Jena Germany; ^5^ Department of Neurology Jena University Hospital Jena Germany; ^6^ German Center for Mental Health (DZPG); ^7^ Department of Neuroradiology University Hospital Tübingen Tubingen Germany; ^8^ Department of Neonatology University Children's Hospital Tübingen Tubingen Germany; ^9^ Institute For Medical Virology and Epidemiology of Viral Diseases University Hospital Tübingen Tubingen Germany

**Keywords:** MRI surface analyses, MRI volumetric analyses, postnatal human Cytomegalovirus infection, premature birth

## Abstract

**Purpose:**

Congenital infection with *human Cytomegalovirus* (hCMV) is a common cause of severe neurodevelopmental disability, while postnatal infection of a term‐born infant will usually not lead to an adverse neurodevelopmental outcome. In preterm‐born infants, long‐term consequences of an early postnatal hCMV infection (usually via breast milk) are still controversial. This is highly relevant as preventative measures exist.

**Methods:**

Data of 37 preterm‐born children (PT; ≤ 32 weeks of gestation and/or weighing ≤ 1500 g) was included. Of these, 14 acquired an early postnatal infection with hCMV (PT_ hCMV+_), while 23 did not (PT_ hCMV−_). Further, 38 healthy term‐born participants (FT) were included. Overall median age was 13.6 years (range 7.9–17.8 years). Global and local tissue volumes and brain surface parameters were analyzed. Consequences of prematurity were detected by comparing FT and PT, and sequelae of hCMV infection by comparing PT_ hCMV−_ and PT_ hCMV+_.

**Findings:**

Compared to FT, PT showed lower global gray matter (GM); interestingly, PT_ hCMV+_ showed a trend toward higher global GM than PT_ hCMV−_. Several clusters of local GM differed in volume between PT and FT, but none as a function of hCMV infection. Surface analyses between PT and FT identified predominantly right‐hemispheric regions of lower cortical thickness in PT. Unexpectedly, widespread clusters of higher cortical thickness were found bilaterally in predominantly frontal brain regions in PT_ hCMV+_ compared to PT_ hCMV−_, demonstrating a lasting effect of hCMV infection.

**Conclusion:**

We found lower global and local GM volumes due to of prematurity. Additionally, we demonstrate long‐term effects of early postnatal hCMV infection on brain structure in PT, markedly different from those resulting from prematurity alone. This suggests distinct long‐term cerebral consequences of early postnatal hCMV infection in former preterm‐born children above and beyond those attributable to prematurity. Consequently, efforts to avoid HCMV infection in preterm‐born infants should be implemented.

## Graphical abstract

Cortical surface analyses of former preterm‐born children with (PT_ hCMV+_) and without (PT_ hCMV−_) early postnatal *human Cytomegalovirus* (hCMV) infection at ∼13 years of age. Note the strong effect of prematurity when compared to full‐term‐born children (FT, top), but the independent and even more widespread effect of HCMV infection (bottom).

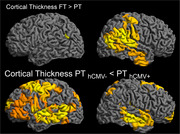

## 1 Introduction

Around 11% of children are born prematurely, i.e., before 37 weeks of gestation. Among these, babies with a low birthweight (< 1500 g) and/or those born before 32 weeks in particular are at considerable risk for early morbidity and mortality. (Stoll et al. 2010, Scheuchenegger et al. 2014, Schill et al. 2017) Importantly, they also carry a high risk for later neurodevelopmental impairment (Moster et al. 2008, Johnson and Marlow 2017, Raju et al. 2017), substantially contributing to the overall burden of disease. (Harrison and Goldenberg 2016) While reasons for preterm birth are diverse, many are impossible to avoid; therefore, preterm birth continues to pose substantial medical and societal challenges.

*Human cytomegalovirus* (hCMV) is among the single most important causes for mental retardation if it leads to intrauterine, congenital infection. (Boppana et al. 2013) Prevalence of congenital hCMV infection was estimated to be 0.5%–1% of live births in industrialized nations (de Vries et al. 2011, Naing et al. 2016) and up to 6% in developing countries. (Lanzieri et al. 2014) In these cases, intrauterine infection occurs via vertical transmission of hCMV from mother to child. In the first and second trimesters, approximately one‐third of primary maternal hCMV infections lead to congenital hCMV infection. Transmission rates increased over 70% in the third trimester. (Enders et al. 2011) Traditionally, only primary infection of hCMV in pregnant women was considered to cause congenital hCMV infection, whereas non‐primary infection during pregnancy was considered to be mostly harmless. (Kenneson and Cannon 2007) More recent research, however, suggested that congenital HCMV infection following non‐primary infection of already seropositive mothers may be as prevalent as primary infection. (Townsend et al. 2013, Wang et al. 2011, Britt 2015) On the other hand, the infection is widely assumed to be of little relevance in term‐born children infected postnatally: the majority of full‐term born infants neither showed clinical symptoms while shedding hCMV nor did they develop long‐term neurologic sequelae. (Gentile et al. 1989, Kumar et al. 1984, Paryani et al. 1985, Johnson et al. 1986, Stagno and Cloud 1994, Schleiss 2006) However, there is considerable uncertainty with regard to the long‐term effects of a postnatal infection in early preterms, occurring *ex utero* but before the expected delivery date. The most common route of infection is via breast milk. The rate of hCMV secretion in breast milk of hCMV‐seropositive mothers to preterm‐born infants ranged up to 96% (Hamprecht et al. 2001, Kurath et al. 2010), and 19% (95% CI 11–32) of the preterm‐born infants exposed to hCMV positive breast milk were infected by hCMV postnatally. (Lanzieri et al. 2013) This rate of transmission depends on the viral load, the duration of viral shedding in breast milk and the time of observation. (Jim et al. 2009, van der Strate et al. 2001) Since most maternal antibodies are transmitted to the fetus only in the third trimester, (Simister 2003) most preterm born infants, especially those born very preterm, are not as effectively protected against postnatal hCMV infection as term born infants are. While the majority of preterm born infants infected with hCMV did not develop clinical symptoms (Neuberger et al. 2006), rates of symptomatic hCMV infection varied greatly between studies, with numbers ranging from 0% up to more than 30%. (Kurath et al. 2010, Lanzieri et al. 2013, Yeager et al. 1983, Miron et al. 2005, Josephson et al. 2014, Bryant et al. 2002) Several cases of severe postnatal hCMV infections in this population have been described, with a wide range of clinical manifestations. In some preterm‐born infants, a fatal outcome was reported. (Yeager et al. 1983, Stagno et al. 1981, Hamele et al. 2010, Takahashi et al. 2007, Fischer et al. 2012, Anne‐Aurelie et al. 2016)

When assessing the early clinical outcome of preterm‐born children with early postnatal hCMV infection at 12 and 24 months, there was no significant impairment. (Jim et al. 2015, Vollmer et al. 2004) This changed at school age: The Tübingen group of former preterm children with early postnatal hCMV infection scored significantly lower in cognitive and motor function tests than controls without such an infection. (Bevot et al. 2012, Goelz et al. 2013) Further, both lower than average intelligence and differences in functional MRI activation patterns could be seen in adolescence (Brecht et al. (2015) and Dorn et al. (2014), assessing children of the same original cohort as presented here). The question of these long‐term effects is crucial against the background that, as breast milk is the usual route of infection, such infections could be prevented by inactivating the virus. This can be achieved for example by short‐term heat inactivation of breast milk in the neonatal intensive care unit. (Hamprecht and Goelz 2017, Maschmann et al. 2019, Bapistella et al. 2019) If further evidence for detrimental long‐term effects could be demonstrated, this could support the discussions with the potential consequence to more broadly implement these preventative measures.

We here set out to investigate a group of former preterm‐born children with and without postnatal hCMV infection and full term‐born children as controls, using both volumetric and surface‐based MRI analyses. The rationale for combining both approaches was that while volumetric analyses such as voxel‐based morphometry (VBM) assess regional tissue volume, surface‐based approaches allow assessing other morphological features, such as cortical thickness. The latter describes the distance between the inner (white matter (WM) / grey matter (GM)) and the outer (GM/cerebrospinal fluid (CSF)) cortical boundaries. It reflects the thickness of grey matter and, on a cellular level, the neuron column's height throughout the cortex. (Rakic 1995) As volumetric and surface parameters show different developmental patterns throughout childhood, it was postulated that each parameter at least partly describes different underlying neuronal processes. (Wierenga et al. 2014, Vijayakumar et al. 2016) Therefore, alterations in brain development may be missed when utilizing only one approach. (Wierenga et al. 2014) The aim of the current study, therefore, was to comprehensively assess whether long‐term structural consequences of an early postnatal hCMV infection are detectable in the brains of former very preterm‐born children in late childhood and adolescence.

## 2 Subjects and Methods

Participants of the present work were part of a long‐term follow‐up study investigating the consequences of an early postnatal infection with hCMV via breast milk in preterm‐born participants. Some of the participants included in this work were also part of previous studies. (Bevot et al. 2012, Goelz et al. 2013, Brecht et al. 2015, Dorn et al. 2014) All participating preterm‐born children (PT) were born between July 1995 and September 1999 and were treated in the NICU at the Children's Hospital of the University of Tübingen. Participants were born at ≤ 32 weeks of gestation and/or weighed ≤ 1500 g at birth. The study was approved by the Ethics Committee of the University of Tübingen Faculty of Medicine (2016/2009BO1). Written informed consent was obtained from at least one parent, and all children additionally gave verbal assent.

In all preterm participants, congenital hCMV infection had been excluded by examining ear and throat swabs and urine in the first postpartum days. (Hamprecht et al. 2001) To detect early postnatal hCMV infection, participants’ urine as well as their mothers' breast milk was examined regularly. If HCMV was detected in the participants’ urine within two to twelve weeks of life, those participants were defined as having acquired an early postnatal infection with HCMV. Thus, they were assigned to group “preterm hCMV+” (PT _hCMV+_). Preterm participants who did not suffer from an early postnatal infection with hCMV are consequently considered “preterm hCMV‐” (PT _hCMV_−). For further information on the virological surveillance procedure see Hamprecht et al. (2001) All former preterm‐born participants were identified from hospital records of the Department of Neonatology and approached in writing. A total of 94 PT were considered for inclusion; 50 hCMV− and 44 hCMV+. Full‐term born controls (FT) were recruited by public announcements. They were required to have no history of neurological or psychiatric disorders, hearing deficits, or cognitive impairment. While their HCMV status was not available, a history of any neonatal infection, hepatosplenomegaly, thrombocytopenia, or prolonged jaundice was also considered an exclusion criterion. Further, all participants were required to have no MR contraindications. (Brecht et al. 2015, Dorn et al. 2014, Wilke et al. 2014) Subjects were divided into two groups: FT and PT, which were again divided into PT _hCMV−_ and PT _hCMV+_, respectively.

Sex, age, and maternal education (ME) were collected from all participants. ME was shown to be a relevant factor for the cognitive outcome of preterm‐born children (Patra et al. 2016) and was scaled to reflect years of the mother's education (9: minimal schooling; 10: regular degree; 13: graduated from German high school); 3 years were added for vocational training and 5 years for a university degree, such that final numbers range from 9 to 18. Additionally, information on relevant neonatal information for the PT group (gestational age, birth weight, singleton or multiple, incidence of intraventricular hemorrhage (IVH), bronchopulmonary dysplasia (BPD), retinopathy of prematurity (ROP), or necrotizing enterocolitis (NEC)) was collected from hospital charts.

Cognitive abilities were assessed by the HAWIK IV (the German version of the Wechsler Intelligence Scale for Children). A standardized full‐scale IQ was obtained. (Petermann and Petermann 2008) The presence of cerebral palsy (CP) was assessed clinically, while handedness was determined by the Edinburgh Handedness Inventory (EHI). (Oldfield 1971)

All participants were scanned at University Hospital Tübingen on the same 1.5 Tesla MR scanner (Siemens Avanto, Erlangen, Germany) with a 12‐channel head coil. Structural T1‐weighted 3D datasets were acquired consisting of 176 sagittal slices of 1 mm thickness with a matrix size of 256 × 256 and no gap, yielding a voxel size of 1 × 1 × 1 mm^3^. Echo time was 2.92 s, and repetition time was 1300 ms. Data was preprocessed using SPM12 (RRID:SCR_007037) (Wellcome Trust Centre for Neuroimaging, University College London, UK) running on MATLAB (R2014b, The Mathworks, Natick) and CAT12 (RRID:SCR_019184), a computational anatomy toolbox. (Gaser and Kurth 2016, Gaser et al. 2024)

All MR images were initially inspected visually, and those with clear subject motion artifacts were removed. As a second step, data quality was checked with the help of CAT12 by creating a sample correlation matrix. In this way, data that deviated substantially from the whole sample was identified. Additionally, CAT12's image quality rating (IQR) was assessed, which combines contrast to noise ratio, an inhomogeneity to contrast ratio, and the root mean square of the image resolution; the lower the IQR, the better the image quality. (Gaser and Kurth 2016, Gaser 2018) This measure allows for ensuring comparability of image quality between groups, thus avoiding bias. Of 83 initial MRI datasets, eight needed to be excluded during these data quality steps due to motion artifacts, leaving 75 datasets for final analyses: 37 in group PT (of which 14 were PT _hCMV+_ and 23 were PT _hCMV_−) and 38 in group FT. More details are provided in Tables 1, 2, and 3 in the results section.

**TABLE 1:** **Demographic and neuropsychological details** of FT versus PT participants.

	FT	PT	Statistics
*n*	38	37	
Sex	M: 16; F: 22	m: 26; f: 11	*n.s*.^1^
Median age (range) [years]	12.1 (7.9–17.8)	14.9 (12.0‐16.1)	*p* < .001^2^
Handedness	87% r; 13% l	81% r; 19% l	*n.s*.^1^
Median ME	15	13	*p* = 0.003^2^
Median IQ (range)	110 (91–128)	97 (42‐137)	*p* < 0.001^2^
Median IQR (range)	2.44 (2.08–4.05)	2.39 (2.08‐3.64)	*n.s*.^2^

**Abbreviations**: FT = full term born participants, IQ = intelligence quotient; PT = preterm born participants; IQR = image quality rating, ME = maternal education.

^1^ Chi‐squared test (Scheuchenegger et al. 2014).

^2^ Mann–Whitney *U* test.

**TABLE 2:** **Demographic and neuropsychological details of preterm born participants** with early postnatal hCMV‐infection (PT _hCMV+_) versus preterm born participants without early postnatal _hCMV−_ infection (PT _hCMV−_).

	PT _hCMV−_	PT _hCMV+_	Statistics
n	23	14	
Sex	M: 16; F: 7	M: 10; F: 4	*n.s*.^1^
Median age (range) [years]	14.9 (12.1–16.1)	14.8 (12.0–16.1)	*n.s*.^2^
Handedness	83% r; 17% l	79% r; 21% l	*n.s*.^1^
Median ME	13	13	*n.s*.^2^
Median IQ (range)	100 (42–137)	93 (61–119)	*n.s*.^2^
Median IQR (range)	2.39 (2.15–3.64)	2.45 (2.08–3.04)	*n.s*.^2^
Twins n (%)	7 (30.4%)	7 (50%)	*n.s*.^1^
Median gestational age (range) [weeks]	28.1 (24–32)	29.7 (25–32)	*n.s*.^2^
Median birth weight (range) [g]	970 (650–1550)	1272 (630–1870)	*n.s*.^2^
ICH n (%)	5 (21.7%)	2 (14.3%)	*n.s*.^1^
NEC n	0	0	
BPD n (%)	5 (21.7%)	1 (7.1%)	*n.s*.^1^
ROP n (%)	10 (43.5%)	3 (21.4%)	*n.s*.^1^

**Abbreviations**: aME = maternal education, BPD = bronchopulmonary dysplasia, ICH = intracranial hemorrhage, IQ = intelligence quotient, IQR = image quality rating, NEC = necrotizing enterocolitis, ROP = retinopathy of prematurity.

^1^ Chi squared test.

^2^ Mann–Whitney *U* test.

**TABLE 3:** **Demographic details of included preterm born participants (PT) and non‐included preterm born subjects** (PT) of the original cohort.

	Included PT	Non‐included PT	Statistics
n	37	57	
hCMV + n (%)	14 (37, 8%)	30 (52.6%)	*n.s*.^1^
Sex	m: 26; f: 11	M: 42; F: 15	*n.s*.^1^
Median gestational age (range) [weeks]	28.9 (23.9–32)	28.3 (23.6–32.1)	*n.s*.^2^
Median birth weight (range) [g]	1140 (630–1870)	1070 (490–1700)	*n.s*.^2^
ICH n (%)	7 (18.9%)	9 (15.8%)	*n.s*.^1^
NEC n (%)	0	3 (5.3%)	*n.s*.^1^
BPD n (%)	6 (16.2%)	7 (12.3%)	*n.s*.^1^
ROP n (%)	13 (35.1%)	16 (28.1%)	*n.s*.^1^
Twins n (%)	14 (37.8%)	13 (22.8%)	*n.s*.^1^

**Abbreviations**: BPD = bronchopulmonary dysplasia, hCMV_+_ = with early postnatal hCMV infection, ICH = intracranial hemorrhage, NEC = necrotizing enterocolitis, ROP = retinopathy of prematurity.

^1^ Chi squared test.

^2^ Mann–Whitney *U* test.

To ensure optimal starting estimates for later data processing steps, all images were reoriented manually, and the image volume “origin” was set to the anterior commissure. For initial segmentation and spatial normalization, we used the Template‐O‐Matic toolbox (Wilke et al., 2008) to create a pediatric template, using a “matched pairs” approach. Images were then segmented into GM, WM, and CSF using CAT12, which employs a revised version of SPM12's unified segmentation approach. (Ashburner and Friston 2005) From the affine‐registered images from this step, we created a custom DARTEL template. (Ashburner 2007) For the final normalization and segmentation of the 75 datasets, the CAT12 processing steps described above were repeated using a newly generated TOM template from the final participants and the study‐specific DARTEL template. To account for local volume changes due to non‐linear spatial registration, we modulated the final maps using the Jacobian determinant of the spatial deformation field. This iterative procedure ensures that results were obtained with a minimum amount of bias from an inappropriate (adult) reference population.

For VBM analyses, a Gaussian smoothing kernel with a “full width at half maximum” (FWHM) of 6 mm was applied. Individual global tissue volumes were derived from the respective modulated tissue maps, including gray matter volume (GM), white matter volume (WM), and cerebrospinal fluid volume (CSFV), which combined yield total intracranial volume (TIV). (Malone et al. 2015) Cortical thickness was obtained by using CAT12. These surface parameters require a larger smoothing kernel, so we used a Gaussian filter of FWHM = 20 mm for these analyses, exploiting the matched filter theorem based on the average distance between sulci and gyri. (Luders et al. 2006)

When aiming to analyze local differences in brain structure using VBM, correction for global differences is a necessary prerequisite (Malone et al. 2015, Barnes et al. 2010, Joel et al. 2015, Peters et al. 1998), which was achieved here by global scaling. Global scaling divides each voxel by the global mean to detect only local effects that exceed (and thus, are not explained by) global differences. Images were proportionally scaled to a value of 50. After scaling, the absolute unscaled threshold for including a voxel (default 0.1) was adjusted as follows: new threshold = (0.1 × 50)/TIV.

As subject sex is known to influence brain anatomy both globally and locally (Joel et al. 2015, Peters et al. 1998), it was included as a covariate in all GLM analyses. Correction for subject age was necessary because groups differed in median age (FT: 12.1 years, PT _hCMV−_: 14.9 years, PT _hCMV+_: 14.8 years). As age effects on brain volumes are non‐linear (Giedd et al. 1999, Groeschel et al. 2010, Wilke et al. 2007), age (in months) as well as age squared was included in the GLM as covariates.

Statistical analysis of demographic data was conducted in APSS 23 (RRID:SCR_002865 IBM Corporation, Armonk, New York, USA). Due to small sample sizes, chi‐squared tests were conducted to test for group differences of dichotomous, independent variables, that is, sex, handedness, and the occurrence of twin birth, ICH, NEC, BPD, and ROP. Group differences in image quality, age at assessment, gestational age, birth weight, IQ, and maternal education were investigated by applying Mann–Whitney *U* tests. Significance was assumed at *p* ≤ 0.05, and results were Bonferroni‐corrected for multiple comparisons.

Statistical analysis of tissue volumes was also conducted in SPSS 23. Due to small sample sizes, Mann–Whitney *U* tests were used to compare groups FT/PT and PT _hCMV−_/PT _hCMV+_. To assess a possible effect of prematurity, we compared FT and PT. To assess a possible effect of early postnatal hCMV infection in PT, we compared groups PT _hCMV−_ and PT _hCMV+_. Again, significance was assumed at *p* ≤ 0.05, and results were Bonferroni‐corrected for multiple comparisons.

The framework of the general linear model (GLM) (Scott et al. 2014) was used to investigate local GM differences between groups. In VBM analyses, the groups were considered to be independent from each other, and variance was assumed to be unequal in order to be statistically most robust. Global differences were corrected by global scaling, as described above. Age, age squared, and sex were included as covariates of no interest. To control for multiple testing in surface‐based analyses, the “Threshold Free Cluster Enhancement” (TFCE) and an additional correction via the “Family Wise Error Rate” (FWE) correction was applied (Salimi‐Khorshidi et al. 2011, Smith and Nichols 2009, Pernet 2016, Li et al. 2017), using 5000 permutations per contrast. To further protect from false positive results, FWE correction on a cluster level (FWE_C_) of *p* ≤ 0.05 was applied. (Nichols and Hayasaka 2003)

All results of VBM are shown in neurological convention, i.e., left in the image is left in the brain. All results of surface‐based analyses are shown rendered on a representative individual cortical surface.

## 3 Results

### 3.1 Demographics

We initially approached 94 preterm‐born children, 44 of whom could be recruited. Four of these had MR contraindications (dental braces, metal splinter). Hence, MR images of 40 preterm children could be acquired, among them 23 hCMV_−_ and 17 hCMV_+_, with a median age of 14.9 years (range 12.0–16.1). For the control group, images of 43 full‐term‐born children, with a median age of 12.1 years (7.9–17.8), were acquired. Images from eight participants were excluded during quality control (3 PT, 5 FT). Consequently, data of 75 images was included in the following analyses: 38 FT and 37 PT (23 PT _hCMV−_ and 14 PT _hCMV+_). There were 42 boys and 33 girls, and the overall median age was 13.6 years (range 7.9–17.8 years). Demographic data and details of neuropsychological assessment are shown below. Groups FT and PT showed significant differences in age, ME, and IQ, but not in sex, handedness, and image quality rating (IQR; Table 1).

Groups PT _hCMV−_ and PT _hCMV+_ did not differ significantly in sex, age, handedness, ME, IQ, IQR, number of twins, gestational age, birth weight, or occurrence of ICH, NEC, BPD, and ROP (Table 2).

To rule out a selection bias, demographic details of the ultimately included preterm born participants (*n* = 37) were compared to all preterms we approached (*n* = 94) to ensure that our sample was representative of the cohort as a whole. Of those 94 PT, 24 did not reply, six did not have time, one suffered from claustrophobia, eight had metallic implants, and 15 provided no reason. Following inclusion, imaging data from three preterm participants had to be excluded. Details of the groups are listed in Table 3. Neither of the assessed differences was significant.

### 3.2 Global Tissue Volumes

Results are summarized in Tables 4 and 5. Mann–Whitney *U* tests were conducted, and significance was assumed at *p* < 0.05, Bonferroni‐corrected for multiple comparisons.

**TABLE 4:** Tissue volumes of full term (FT) versus preterm born participants (PT).

	FT	PT	Statistics
Mean TIV ± SD [ml]	1653 ± 157	1578 ± 164	*n.s*.
Mean GM ± SD [ml]	855 ± 76	783 ± 73	*p* < 0.001
Mean WM ± SD [ml]	522 ± 64	504 ± 74	*n.s*.
Mean CSF ± SD [ml]	276 ± 41	290 ± 39	*n.s*.

**Abbreviations**: CSF = cerebrospinal fluid volume, GM = gray matter volume; TIV = total intracranial volume; WM = white matter volume.

**TABLE 5:** **Tissue volumes of preterm born participants** with (PT _hCMV+_) and without (PT _hCMV‐_) postnatal hCMV infection.

	PT _hCMV+_	PT _hCMV−_	Statistics
Mean TIV ± SD [ml]	1599 ± 162	1565 ± 167	*n.s*.
Mean GM ± SD [ml]	802 ± 80	771 ± 68	*n.s*.
Mean WM ± SD [ml]	510 ± 67	500 ± 81	*n.s*.
Mean CSF ± SD [ml]	286 ± 36	293 ± 41	*n.s*.

**Abbreviations**: CSF = cerebrospinal fluid volume, GM = gray matter volume, TIV = total intracranial volume; WM = white matter volume.

### 3.3 Global Tissue Volumes: Effects of Prematurity

Comparison of FT and PT showed no significant difference in TIV. PT showed a significant decrease in GM as compared to FT. In post hoc targeted comparisons, the difference between FT and PT was driven by the PT _hCMV−_ group, which had a significantly lower GM than FT (while no significant difference in GM was found when comparing FT to PT _hCMV+_). Comparison of PT versus FT showed no significant difference in WM or CSF volumes.

### 3.4 Global Tissue Volumes: Effects of hCMV Infection

Comparison of PT _hCMV+_ versus PT _hCMV−_ showed no significant difference in TIV. Comparison of PT _hCMV−_ versus PT _hCMV+_ showed no significant difference in GM, but contrary to our hypothesis, PT _hCMV+_ had a tendency toward higher GM than PT _hCMV‐;_ however, this difference was not significant. Comparison of PT _hCMV+_ versus PT _hCMV−_ showed no significant difference in WM or CSF volumes.

### 3.5 Voxel Based Morphometry

To assess a possible local effect of prematurity on GM (exceeding global differences), we performed VBM studies between groups FT and PT. A GLM was designed, correcting for sex and age (linear and squared) and for TIV differences by global scaling. Significance was assumed after applying TFCE and correcting via FWEc (*p* ≤ 0.05).

### 3.6 VBM: Effects of Prematurity

**FIGURE 1:**
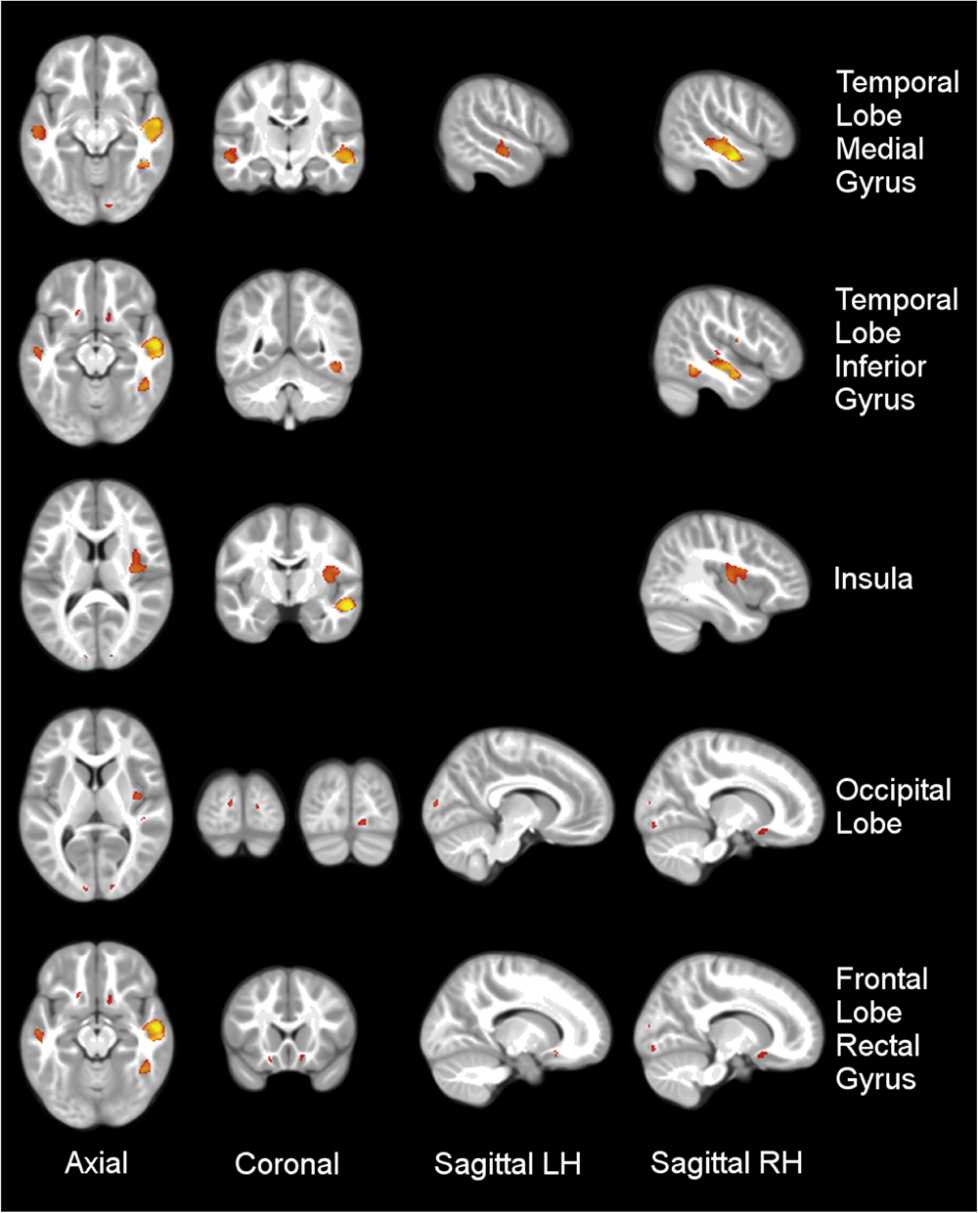
**Comparison of local GM: FT versus PT participants**. PT showed decreased local GM in the left and right medial temporal gyrus, the right inferior temporal gyrus, the left and right occipital lobe, the left and right middle orbitofrontal gyrus and the right insula. Clusters are overlaid on the normalized average T1 image of the study group. Results are shown in neurologic convention. (TFCE, FWE *p* < 0.05.) **Abbreviations**: LH = left hemisphere, RH = right hemisphere.

PT showed significantly lower local GM than FT (FT > PT) in the medial temporal gyrus in both hemispheres, in the middle orbitofrontal gyrus in both hemispheres, in the occipital lobe in both hemispheres, in the inferior temporal gyrus in the right hemisphere, and in the medial insula in the right hemisphere. Results are visualized in Figure 1. PT showed no significant cluster of increased local GM compared to FT (FT < PT).

### 3.7 VBM: Effects of hCMV infection

There were no significant differences in local GM between PT _hCMV+_ and PT _hCMV−_, in either direction.

### 3.8 Cortical Thickness Analyses

Comparisons were corrected for sex and age (linear and squared). Significance was assumed after applying TFCE and correcting via FWEc (*p* ≤ 0.05).

### 3.9 Cortical Thickness Analyses: Effects of Prematurity

**FIGURE 2:**
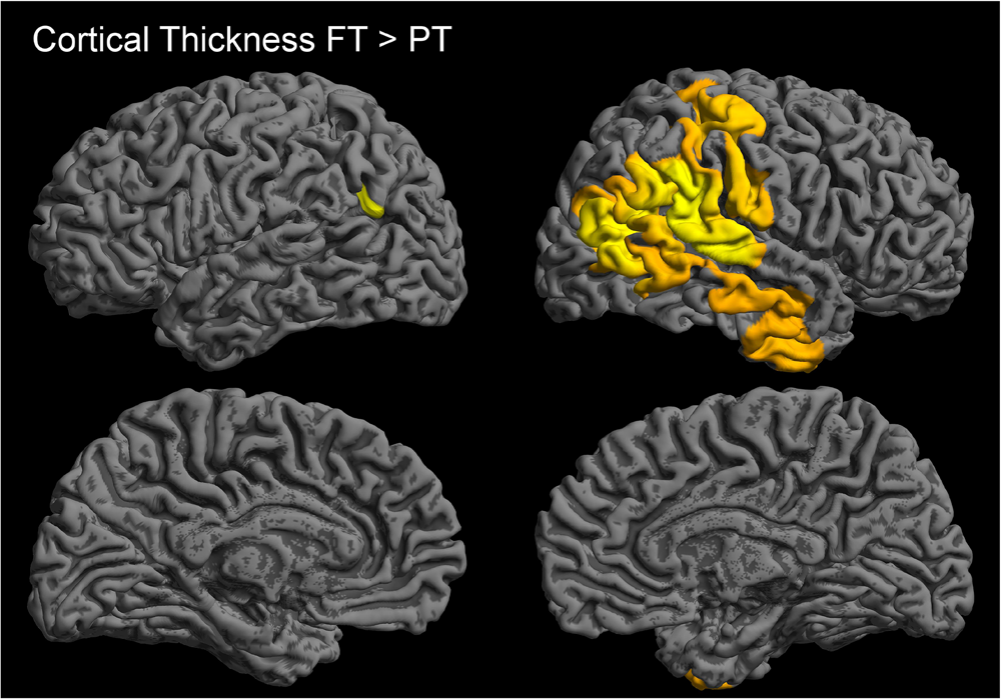
**Comparison of local cortical thickness: FT versus PT participants**. PT showed significantly lower cortical thickness compared to FT, predominantly in temporal and parietal regions of the right hemisphere. In the left hemisphere, only a small area of parietal lobe showed significantly lower cortical thickness of PT compared to FT. (GLM, TFCE, FWE_c_ *p* < 0.05.).

PT showed locally lower cortical thickness predominantly in the right hemisphere, including temporal (superior, medial, and inferior gyrus) and parietal regions (postcentral, supramarginal, and angular gyrus) as well as lateral aspects of the occipital lobe, compared to FT. In the left hemisphere, only a small area in the angular gyrus showed lower cortical thickness in PT. Results are visualized in Figure 2. There were no significant clusters where PT had higher cortical thickness than FT.

### 3.10 Cortical Thickness Analyses: Effects of hCMV Infection

**FIGURE 3:**
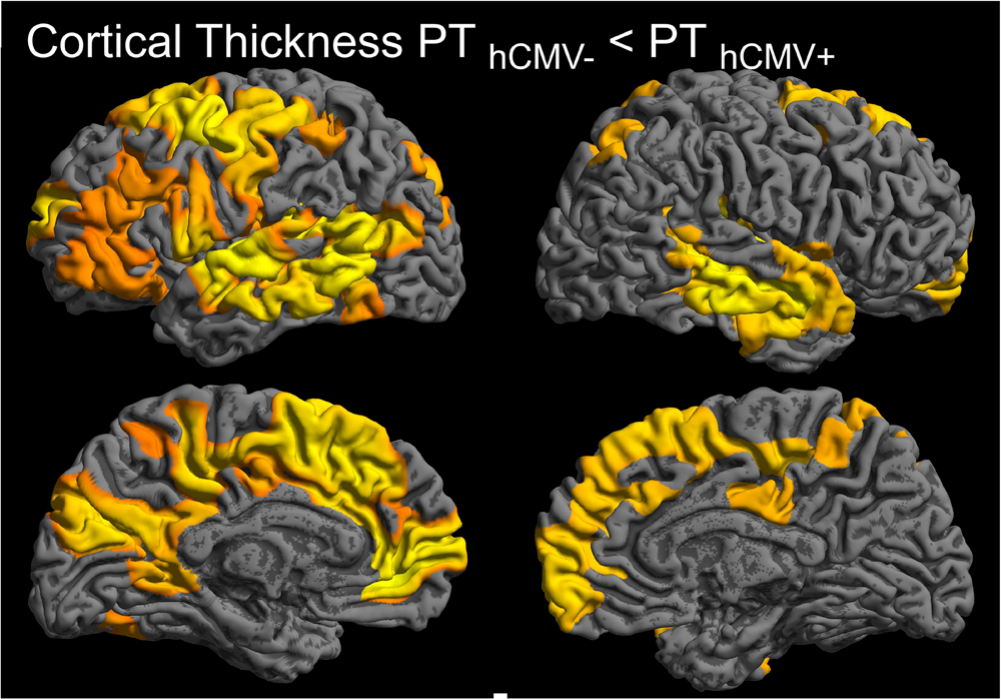
**Comparison of local cortical thickness: PT _hCMV−_ versus PT_ hCMV+_**. PT_ hCMV+_ showed widespread regions of significantly higher cortical thickness compared to PT_ hCMV‐_. This was more pronounced in the left hemisphere, including areas of the temporal, parietal, occipital and frontal lobe. In the right hemisphere, cortical thickness was higher in areas of the temporal, parietal and frontal lobe. (GLM, TFCE, FWE_c_ *p* < 0.05.).

PT _hCMV+_ showed widespread higher cortical thickness when compared to PT _hCMV‐_. Higher cortical thickness was predominant frontally but present in all lobes, more pronounced in the left hemisphere. The left hemisphere showed cluster peaks in the superior and medial temporal gyrus, the frontolateral occipital lobe, the cuneus, the pre‐ and postcentral gyrus, the precuneus and angular gyrus in the parietal lobe, the precentral and superior and inferior frontal gyrus, the frontal pole, and the cingulate. The right hemisphere showed cluster peaks in the superior and medial temporal lobe, superior parietal lobe, superior frontal gyrus, frontal pole, and medial cingular gyrus. Results are visualized in Figure 3. There were no clusters of lower cortical thickness in PT _hCMV+_ compared to PT _hCMV−_.

## 4 Discussion

The aim of this work was to assess the long‐term influence of prematurity in general and of early postnatal hCMV infection in particular on the brain structure of former preterm‐born children in late childhood and adolescence. To address the first question, we compared imaging data from 37 PT participants to data from 38 FT participants. To address the second question, 23 PT _hCMV+_ participants were compared to 14 PT _hCMV−_ participants. We applied volumetric analyses (global and local tissue volumes) as well as surface‐based analyses. We hypothesized that early postnatal hCMV‐infection would have long‐term consequences on brain structure, in addition to the influence of prematurity per se.

### 4.1 Cohort

The finally included PT differed from the FT children in age, which was statistically accounted for, and in maternal education (Table 1). Maternal education, on the one hand, is linked to prematurity per se (Ruiz et al. 2015) but is also known to play an important role in cognitive outcome after prematurity. (Patra et al. 2016) Considering this, the additional difference in IQ between all PT and FT must be discussed critically; however, it is in good agreement with the large body of evidence demonstrating the intelligence of former PT in the low average range. (Raju et al. 2017, Bhutta et al. 2002, Baumann et al. 2015, Hutchinson et al. 2013, Anderson 2014) Hence, our sample's characteristics are generally in line with previous studies on neurocognitive impairment of preterm born children at school age and beyond.

Importantly, we were able to assess a rather large group of excellently characterized very preterm born children treated in only one tertiary care center. The stringent virological screening program established very early on (Hamprecht et al. 2001) allowed us to unequivocally categorize all PT participants as either PT _hCMV−_ or PT _hCMV+_, and to rule out congenital hCMV infection as a potentially important confound. Importantly, these groups did not differ from each other in a number of relevant neonatal characteristics (Table 2). To further rule out a post‐hoc selection bias, we also assessed the original, full cohort that could have participated and found the finally‐included PT to be fully representative (Table 3). These factors suggest a valid basis for the interpretation of our results.

### 4.2 Effects of Prematurity

MRI analyses of global brain volumes showed a tendency of lower TIV in PT than FT, which, however, was only significant when comparing FT to PT _hCMV−_. PT showed significantly lower global GM than FT (the impact of the two PT groups will be discussed below). Those results are in accordance with previous studies showing lower GM at term equivalent age as well as later on. (Monson et al. 2016, Thompson et al. 2007, Padilla et al. 2015) Differences in GM between preterm‐born and full‐term‐born children even increased throughout childhood. (Monson et al. 2016) Discrepancies may have resulted from a higher rate of ICH, WM injury, and neonatal complications in other published preterm‐born groups. (Monson et al. 2016, Thompson et al. 2007) Reduced GM in infancy and childhood was associated with lower scores in IQ testing in the literature (Monson et al. 2016), which is in line with our observations in this sample.

VBM analyses revealed that local GM (irrespective of global differences) of PT was lower predominantly in temporal lobes and the right insula. Reduced GM in the medial temporal gyrus, as was apparent in our PT group compared to FT, has been reported many times in groups with a history of premature birth, across a wide age range (nine‐year‐olds (Soria‐Pastor et al. 2009, Zubiaurre‐Elorza et al. 2011) male 12‐year‐olds (Kesler et al. 2008), adolescents (Nosarti et al. 2008), and adults). (Bauml et al. 2015) Furthermore, our analyses showed lower local GM in the right inferior temporal gyrus in PT compared to FT. Lower GM in the inferior temporal gyrus has been reported previously in preterm‐born young adults; interestingly, also only in the right hemisphere in both studies. (Bauml et al. 2015, Nosarti et al. 2014) Likewise, regional brain volume measurement in preterm‐born infants at term‐equivalent age revealed lower volume of the right inferior temporal gyrus. (Gousias et al. 2012) The temporal lobe is known to play a role in language processing (Helenius et al. 2014, Marslen‐Wilson and Tyler 1980), reading acquisition (Monzalvo and Dehaene‐Lambertz 2013), working memory (Jeneson and Squire 2012), and learning. (Dalton et al. 2016) Further, studies have previously reported an association of altered temporal lobe structure with cognitive abilities. In preterm‐born young adults, lower GM in the medial temporal gyrus was associated with lower executive function scores. (Nosarti et al. 2014) Hence, structural alterations of the temporal lobe may play a role in impairment of cognitive, reading, speech, and learning abilities, all of which have been described in preterm‐born children. (Hutchinson et al. 2013, Bowen et al. 2002, Aarnoudse‐Moens et al. 2011) Overall, we see our results in line with findings indicating enhanced vulnerability of the temporal lobe in preterm‐born children.

Moreover, the insula of the right hemisphere showed lower local GM in VBM analyses, in line with previous studies in adolescents and in young adults with a history of preterm birth. (Nosarti et al. 2008, Nosarti et al. 2014) The insula functions as an integrating center of various networks and, as part of the limbic system, plays a role in sensory and motoric networks, distinguishes between salient and irrelevant impulses from internal and external stimuli, influences attention, focuses working memory, and plays a role in human consciousness. (Menon and Uddin 2010, Augustine 1996, Craig 2009)

Analyses of local GM further revealed lower GM also in the bilateral middle orbitofrontal cortex in PT compared to FT. Structural abnormalities in this region have been described previously in preterm‐born individuals at term‐equivalent age as well as in adults. (Ball et al. 2012, Gimenez et al. 2006) Patients with damage to the orbital prefrontal cortex had difficulties in gambling tasks and in choices between actions and in planning ahead. (Rogers et al. 1999, Bechara 2004) While these may be behavioral manifestations of the structural alterations observed here, the ultimate clinical relevance of structural alterations of the middle orbitofrontal cortex is yet unclear.

Last, PT compared to FT showed lower GM in small clusters located in the occipital lobe, again already described in preterm‐born adolescents and young adults. (Nosarti et al. 2008, Nosarti et al. 2014) The clusters, though small, are located in the extrastriate visual cortex, which plays a role in object, face, and number recognition, spatial attention, and visuospatial information. (Allison et al. 1994, Desimone 1998) Visuospatial problems are widespread in former preterm‐born individuals, especially in connection with posterior WM lesions. (Pavlova et al. 2003, Pavlova et al. 2007) These problems may be enhanced by additional GM lesions within the same system, but no analyses were done here to correlate possible visuospatial impairments in our subjects with these volume reductions in the extrastriate cortex.

Cortical thickness was lower in PT, predominantly in circumscribed regions of the superior and middle temporal lobe, interestingly only in the right hemisphere. Lower cortical thickness in the temporal lobe has already been reported in 15‐ and 20‐year‐old teenagers with low birth weight (Bjuland et al. 2013, Martinussen et al. 2005), in the left temporal lobe of 7–12 year‐old preterm born children (Murner‐Lavanchy et al. 2014), in the bilateral middle and inferior temporal lobe of 15‐year‐old preterm‐born individuals (Nagy et al. 2011), and in 16‐year‐old preterm‐born teenagers. (Frye et al. 2010) Bjuland and colleagues (2013) further reported an association of lower cortical thickness in the temporal lobe with the perceptual organization index of IQ testing, though not full‐scale IQ. Hence, our findings in cortical thickness are also well in line with the effect of prematurity on the brain, even in late childhood and adolescence.

### 4.3 Previous Research on the Effect of Postnatal hCMV Infection in Brain Structure

The main objective of this work was to analyze the consequences of early postnatal hCMV infection on the brain structure of preterm‐born children. We hypothesized that hCMV would have long‐term consequences on brain structure, in addition to the influence of prematurity.

So far, only a few studies have focused on the brain structure of preterm‐born children with early postnatal hCMV infection in adolescent age. One study at term‐equivalent age showed a higher incidence of lenticulostriate vasculopathy in preterm‐born infants with early postnatal hCMV using ultrasound. (Nijman et al. 2012) These children further showed lower fractional anisotropy in occipital WM, indicating microstructural WM alterations (Nijman et al. 2013), whereas in a recent study, also at term equivalent age, no microstructural changes were found in a small group of preterm born infants with and without postnatal hCMV infection. (Pellkofer et al. 2023) In preterm‐born infants, PVL and subsequent WM damage are considered to be the main driving pathology leading to cortical structural alterations. (Volpe 2009) It has been suggested that congenital HCMV infection shows a similar pattern of white matter affection as does PVL. (van der Voorn et al. 2009) These findings would support the theory that in postnatal hCMV infection, at least part of the cortical alterations are due to an underlying WM damage, as suggested before for more typical preterm brain WM lesions. (Volpe 2009, Inder et al. 2023) The prominent role of inflammation may be another similarity, as discussed below.

### 4.4 Effects of Early Postnatal hCMV Infection in Former Preterm Infants

As expected, analyses of global brain volumes revealed that PT _hCMV+_ showed a tendency of lower TIV and GM compared to FT controls. Surprisingly, PT _hCMV+_ showed a tendency toward *greater* TIV and GM when compared to PT _hCMV−_. Assuming a detrimental impact of postnatal HCMV infection, our expectation was the inverse effect. Counterintuitively, the difference in global volumes was therefore less pronounced between PT _hCMV+_ and FT than between PT _hCMV−_ and FT. Cortical volume is directly dependent on cortical thickness and surface area (Wierenga et al. 2014, Raznahan et al. 2011), of which only cortical thickness was investigated in this study. Considering the differences in cortical thickness described below, we suggest that the mechanism that results in more global GM in PT _hCMV+_ is the higher cortical thickness over large parts of the brain in PT _hCMV+_ compared to PT _hCMV−_, as demonstrated by our surface‐based analyses (see below). Thus, the counterintuitively higher volume of GM in PT _hCMV+_ would indirectly reflect widespread structural alterations in cortical thickness.

In line with this hypothesis, analyses of local GM did not yield any significant clusters when comparing PT _hCMV−_ to PT _hCMV+_. Since various authors have reported an influence of prematurity on local GM as discussed above, an additional effect of hCMV on local GM had seemed likely *a priori*. However, local GM, cortical surface, and cortical thickness are the product of at least partly independent processes during brain development (Wierenga et al. 2014); hence, an effect on the one (cortical thickness) would plausibly lead to differences in global, but not necessarily local, tissue volumes. It is also noteworthy that we used global scaling to correct for global tissue volume differences; hence, our VBM approach would only find local tissue volume differences above and beyond global differences.

Interestingly, there were widespread clusters of higher cortical thickness in PT _hCMV+_, predominantly in the frontal, temporal, and parietal lobes of both hemispheres, with a left dominance. This demonstrates that early postnatal hCMV infection in PT affected various parts of the brains, which still resulted in higher cortical thickness at the age of assessment (∼13.6 years later). Since PT _hCMV+_ were compared to PT _hCMV−_, these differences were not a consequence of prematurity per se. As described above, many other neonatal risk factors were also balanced between the two groups, suggesting that these differences in cortical thickness can be specifically attributed to the postnatal hCMV infection.

Previous research has shown that development of cortical thickness, at least in the first year of life, was determined by cortical thickness at birth (Meng et al. 2017), indicating that cortical thickness was a sensitive parameter for early cortical alterations in the form of disturbed intra‐cortical organization. However, they could also represent secondary alterations, reflecting a delay in brain maturation. Both hypotheses will be discussed below.

### 4.5 Possible Mechanism of Early Brain Damage by Early Postnatal hCMV Infection in PT

From a purely chronological standpoint, early postnatal hCMV infection in preterm‐born infants equates to congenital hCMV infection in the third trimester. In contrast to infections in the first and second trimesters of pregnancy, which can cause microcephaly, lissencephaly, and polymicrogyria, infections in the third trimester were traditionally described as having no macroscopic influence on brain morphology. (Barkovich and Lindan 1994) Moreover, while it was suggested that intrauterine hCMV infections in the third trimester were not associated with long‐term sequelae (Enders et al. 2011, Foulon et al. 2008, Oosterom et al. 2015, Chatzakis et al. 2023), PT with early postnatal hCMV infection showed cognitive impairment in previous studies. (Bevot et al. 2012, Goelz et al. 2013, Brecht et al. 2015) Compared to fetuses, preterm‐born infants additionally must cope with the strenuous extrauterine environment and organ‐immaturity‐related comorbidities. Furthermore, quantitatively the largest amounts of maternal antibodies are transmitted via the placenta only during the third trimester. (Simister 2003) Therefore, preterm‐born infants undergoing this infection lack passive maternal immunoprotection. For all those reasons, it seems likely that preterm‐born infants are at a higher risk of an adverse outcome from early postnatal hCMV infection as compared with fetuses suffering from a congenital hCMV infection in the third trimester, although principally of the same age.

The pathogenesis of early postnatal HCMV infection on a microscopic level has not yet been investigated. In contrast, the impact of hCMV in congenital infection is well described. Here, four main pathomechanisms were hypothesized to disrupt brain development: damage to stem cells (Teissier et al. 2014), impaired cell migration into the cortex (Cheeran et al. 2009), damage to glial cells (Cheeran et al. 2009) and detrimental effects of the host's immune response. (Gabrielli et al. 2012) When discussing the potential relevance of these in early postnatal hCMV infection, some seem more likely than others. With regard to the first two, the main body of stem cells has already differentiated in preterm‐born infants, and cellular migration into the cortex is mostly complete at 24 weeks of gestation (Volpe 2009); hence, these two pathomechanisms seem less likely to be crucial in postnatal hCMV infection. More relevant could be the damage to glial cells in early postnatal hCMV infection, since axon formation is still well underway in the third trimester (Kostovic and Judas 2002), and impaired axon development may also have an impact on cortical structure. Finally, systemic inflammatory processes were shown to be detrimental for later cognitive abilities in former preterm‐born toddlers. (Leviton et al. 2013) Cytokines and chemokines secreted in inflammatory processes change the microenvironment in the developing brain and may also alter neural differentiation and migration in congenital hCMV infection. (Cheeran et al. 2009) Taken together and extrapolating from congenital hCMV infection, the long‐term detrimental potential of hCMV is obvious, but the exact underlying pathomechanism can only be speculated upon.

### 4.6 Potential Secondary Impact of Early Postnatal hCMV Infection in PT

In addition to the initial damage to the postnatal brain as a direct or indirect consequence of hCMV infection, altered brain structure in adolescence may also result from altered brain maturation or as a result of compensatory mechanisms.

Previous research on brain development showed that MR scans at birth could predict cortical thickness development in the first year of life with high accuracy. Those findings indicate that dynamics of cortical thickness development, at least in the first year, are determined already at birth. (Meng et al. 2017) Later on and as a function of normal brain maturation, however, cortical thickness decreases in most areas of the brain throughout late childhood and adolescence. (Vijayakumar et al. 2016, Raznahan et al. 2011, Schnack et al. 2015, Aleman‐Gomez et al. 2013) This allows for the interpretation that the here‐observed higher cortical thickness in our school‐aged sample of preterm born children reflects a “lack of the naturally occurring thinning” and thus, a delay in cortical maturation. (Murner‐Lavanchy et al. 2014, Nam et al. 2015) In fact, we could previously show that prematurity is associated with a delay in BrainAGE (Franke et al. 2012), which would be in line with this hypothesis.

While seemingly counterintuitive, higher cortical thickness may be associated with a worse cognitive outcome. In healthy children, cortical thinning over time was associated with higher cognitive abilities in children, such that the more pronounced the cortical thinning was, and the earlier it set in, the higher the IQ was. (Schnack et al. 2015, Shaw et al. 2006) Complementing and consistent with this observation, higher cortical thickness in preterm‐born children was associated with lower IQ at 12 years of age and lower executive function at 15 years of age. (Nam et al. 2015, Brouwer et al. 2014) Hence, a delay in cortical thinning (and thus, relatively higher cortical thickness) may be associated with impaired cognitive abilities of PT _hCMV+,_ in particular, potentially not only reflecting an additional initial insult by hCMV but also an interference with later physiological brain maturational processes.

### 4.7 Strengths and Limitations of this Work

The first limitation of this paper is relatively small sample sizes. Of 94 PT from the original cohort, only 37 were included in the present work. Those 57 not included did not reply, did not want to participate, had contraindications for MRI, or showed movement artifacts in their MR data. However, the PT included in this work did not differ significantly in postnatal clinical data from the whole original cohort and can therefore be considered representative of the original cohort. Further, of the 37 included PT, only 14 were hCMV+. Small samples may not accurately reflect the whole patient cohort they claim to represent. On the other hand, while smaller groups may lack the power to detect subtle differences, the differences that are detected can be taken to be robust (if appropriately controlled for multiple comparisons, as done here).

Conversely, while further subgroup analyses by, e.g., gestational age or other factors would have been desirable, such subgroups become prohibitively small, precluding robust neuroimaging data analyses.

Regrettably, significant differences in age between PT and FT made it necessary to correct for differences in age. Nevertheless, PT _hCMV−_ and PT _hCMV+_ did not differ significantly in age. Analyses between PT _hCMV−_ and PT _hCMV+_ therefore cannot be expected to be driven or mitigated by age differences. Moreover, PT were older than FT and, thus, effectively had “a head start” in brain development, and age was further corrected for by including it as a covariate. If anything, it must be expected that significant differences in age might have led to an underestimation of alterations in brain structure between PT and FT.

To what extent the observed differences in regional volume or in cortical morphology are stable, aggravate, or mitigate over time would require imaging at different timepoints, which regrettably is not available for this cohort. It could also be argued that not all neonatal complications associated with long‐term neurologic sequelae have been included in our preterm cohorts as covariates (Pellkofer et al. 2023); however, ROP as a substitute for critical neonatal courses, such as, for example, sepsis (Jacobson et al. 2021), was not significantly different between our cohorts. Specifically, while no data exists to suggest that CNS infection may have been more prevalent in the one group than in the other, this also cannot be excluded.

Additionally, we have no serologic information on possible postnatal hCMV infections in our term born control children; however, even if present, such infections in the control group would only mitigate the differences between the groups (instead of inflating them). Hence, while a limitation, this lack of information does not invalidate our findings, nor our interpretation.

Due to the increasingly recognized detrimental effects of early postnatal hCMV infection, (Hamele et al. 2010, Fischer et al. 2012, Anne‐Aurelie et al. 2016, Bevot et al. 2012, Goelz et al. 2013, Brecht et al. 2015, Dorn et al. 2014), the original study design could not easily be repeated as more and more centers in Germany move toward pasteurization of breast milk if the hCMV status of the mother is positive. (Buxmann et al. 2010, Klotz et al. 2018) Therefore, our data and results are a unique source of knowledge on the impact of early postnatal hCMV infection on brain structure and neurocognitive abilities in former preterm‐born children.

### 4.8 Summary and Conclusion

In agreement with previous studies, our preterm‐born participants showed decreased global and local GM when compared to full‐term‐born controls. Those findings were supplemented by differences in cortical thickness analyses as a function of prematurity, which have so far not yet been widely reported.

Additionally, hCMV‐infected PT showed widespread clusters of higher cortical thickness compared to non‐infected PT, demonstrating that early postnatal hCMV infection had long‐term consequences on brain structure. The lack of overlap with the brain changes resulting from preterm birth indicates that the effect of hCMV is independent of the influence of prematurity per se. Counterintuitive higher global GM in PT _hCMV+_ could be explained by this widespread higher cortical thickness. This may be a consequence of an early interference with cortical organization and/or of impaired cortical maturation postnatally as a function of early postnatal hCMV infection.

In summary, across several analyses, there were both robust effects of prematurity (differences between FT and PT) and of early postnatal hCMV infection (differences between PT _hCMV+_ and PT _hCMV−_). Remarkably, the patterns of cortical alterations were markedly different from each other, with little overlap, suggesting a distinct and independent impact of either factor. Further, the full extent and some seemingly contradictory effects were only detected and explained by combining different image analysis approaches, which is in line with previous observations. (Wierenga et al. 2014) We believe that these results argue in favor of further implementation of efforts to prevent such an infection in preterm‐born infants.

## Author Contributions

**Meike Müller**: conceptualization, formal analysis, investigation, software, visualization, writing–original draft, writing–review and editing, **Karen Lidzba**: conceptualization, methodology, supervision, writing–review and editing, **Christian Gaser**: conceptualization, formal analysis, methodology, software, writing–review and editing, **Till‐Karsten Hauser**: formal analysis, investigation, writing–review and editing, **Rangmar Goelz**: conceptualization, funding acquisition, investigation, resources, writing–review and editing, **Klaus Hamprecht**: conceptualization, methodology, supervision, and writing–original draft, **Marko Wilke**: conceptualization, formal analysis, funding acquisition, project administration, software, supervision, validation, writing–original draft, writing–review and editing.

## Conflicts of Interest

The authors declare no conflicts of interest.

## Peer Review

The peer review history for this article is available at https://publons.com/publon/10.1002/brb3.70985.

## Data Availability

The MRI data used for this study cannot be shared as it may contain recognizable features and subjects and families were not asked for permission at the time of inclusion on this study.

## References

[brb370985-bib-0001] Aarnoudse‐Moens, C. S. , J. Oosterlaan , H. J. Duivenvoorden , J. B. van Goudoever , and N. Weisglas‐Kuperus . 2011. “Development of Preschool and Academic Skills in Children Born Very Preterm.” Journal of Pediatrics 158, no. 1: 51–56. 20708749 10.1016/j.jpeds.2010.06.052

[brb370985-bib-0002] Aleman‐Gomez, Y. , J. Janssen , H. Schnack , et al. 2013. “The Human Cerebral Cortex Flattens During Adolescence.” The Journal of Neuroscience: The Official Journal of the Society for Neuroscience 33, no. 38: 15004–15010. 24048830 10.1523/JNEUROSCI.1459-13.2013 PMC6618418

[brb370985-bib-0003] Allison, T. , G. McCarthy , A. Nobre , A. Puce , and A. Belger . 1994. “Human Extrastriate Visual Cortex and the Perception of Faces, Words, Numbers, and Colors.” Cerebral Cortex 4, no. 5: 544–554. 7833655 10.1093/cercor/4.5.544

[brb370985-bib-0004] Anderson, P. J. 2014. “Neuropsychological Outcomes of Children Born Very Preterm.” Seminars in Fetal and Neonatal Medicine 19, no. 2: 90–96. 24361279 10.1016/j.siny.2013.11.012

[brb370985-bib-0005] Anne‐Aurelie, L. , B. Souad , and K. Leila . 2016. “Clinical Findings and Autopsy of a Preterm Infant With Breast Milk‐Acquired Cytomegalovirus Infection.” AJP Reports 6, no. 2: e198–202. 27257513 10.1055/s-0035-1566249 PMC4889443

[brb370985-bib-0006] Ashburner, J. 2007. “A Fast Diffeomorphic Image Registration Algorithm.” NeuroImage 38, no. 1: 95–113. 17761438 10.1016/j.neuroimage.2007.07.007

[brb370985-bib-0007] Ashburner, J. , and K. J. Friston . 2005. “Unified Segmentation.” NeuroImage 26, no. 3: 839–851. 15955494 10.1016/j.neuroimage.2005.02.018

[brb370985-bib-0008] Augustine, J. R. 1996. “Circuitry and Functional Aspects of the Insular Lobe in Primates Including Humans.” Brain Research Brain Research Review 22, no. 3: 229–244. 10.1016/s0165-0173(96)00011-2 8957561

[brb370985-bib-0009] Ball, G. , J. P. Boardman , D. Rueckert , et al. 2012. “The Effect of Preterm Birth on Thalamic and Cortical Development.” Cerebral Cortex 22, no. 5: 1016–1024. 21772018 10.1093/cercor/bhr176 PMC3328341

[brb370985-bib-0010] Bapistella, S. , K. Hamprecht , W. Thomas , et al. 2019. “Short‐Term Pasteurization of Breast Milk to Prevent Postnatal Cytomegalovirus Transmission in Very Preterm Infants.” Clinical Infectious Diseases: An Official Publication of the Infectious Diseases Society of America 69, no. 3: 438–444. 30407512 10.1093/cid/ciy945

[brb370985-bib-0011] Barkovich, A. J. , and C. E. Lindan . 1994. “Congenital Cytomegalovirus Infection of the Brain: Imaging Analysis and Embryologic Considerations.” AJNR: American Journal of Neuroradiology 15, no. 4: 703–715. 8010273 PMC8334193

[brb370985-bib-0012] Barnes, J. , G. R. Ridgway , J. Bartlett , et al. 2010. “Head Size, Age and Gender Adjustment in MRI Studies: A Necessary Nuisance?” NeuroImage 53, no. 4: 1244–1255. 20600995 10.1016/j.neuroimage.2010.06.025

[brb370985-bib-0013] Eryigit Madzwamuse S, Baumann, N. , J. Jaekel , P. Bartmann , and D. Wolke . 2015. “Neuro‐cognitive Performance of Very Preterm or Very Low Birth Weight Adults at 26 Years.” Journal of Child Psychology and Psychiatry, and Allied Disciplines 56, no. 8: 857–864. 25382451 10.1111/jcpp.12358

[brb370985-bib-0014] Bauml, J. G. , M. Daamen , C. Meng , et al. 2015. “Correspondence between Aberrant Intrinsic Network Connectivity and Gray‐Matter Volume in the Ventral Brain of Preterm Born Adults.” Cerebral Cortex 25, no. 11: 4135–4145. 24935776 10.1093/cercor/bhu133

[brb370985-bib-0015] Bechara, A. 2004. “The Role of Emotion in Decision‐making: Evidence From Neurological Patients With Orbitofrontal Damage.” Brain and Cognition 55, no. 1: 30–40. 15134841 10.1016/j.bandc.2003.04.001

[brb370985-bib-0016] Bevot, A. , K. Hamprecht , I. Krageloh‐Mann , S. Brosch , R. Goelz , and B. Vollmer . 2012. “Long‐Term Outcome in Preterm Children With *Human Cytomegalovirus* Infection Transmitted via Breast Milk.” Acta Paediatrica 101, no. 4: e167–172. 22111513 10.1111/j.1651-2227.2011.02538.x

[brb370985-bib-0017] Bhutta, A. T. , M. A. Cleves , P. H. Casey , M. M. Cradock , and K. J. Anand . 2002. “Cognitive and Behavioral Outcomes of School‐aged Children Who Were Born Preterm: A Meta‐Analysis.” JAMA 288, no. 6: 728–737. 12169077 10.1001/jama.288.6.728

[brb370985-bib-0018] Bjuland, K. J. , G. C. Lohaugen , M. Martinussen , and J. Skranes . 2013. “Cortical Thickness and Cognition in Very‐low‐birth‐weight Late Teenagers.” Early Human Development 89, no. 6: 371–380. 23273486 10.1016/j.earlhumdev.2012.12.003

[brb370985-bib-0019] Boppana, S. B. , S. A. Ross , and K. B. Fowler . 2013. “Congenital Cytomegalovirus Infection: Clinical Outcome.” Clinical Infectious Diseases: An Official Publication of the Infectious Diseases Society of America 57, no. 4: S178–181. 24257422 10.1093/cid/cit629 PMC4471438

[brb370985-bib-0020] Bowen, J. R. , F. L. Gibson , and P. J. Hand . 2002. “Educational Outcome at 8 Years for Children Who Were Born Extremely Prematurely: A Controlled Study.” Journal of Paediatrics and Child Health 38, no. 5: 438–444. 12354257 10.1046/j.1440-1754.2002.00039.x

[brb370985-bib-0021] Brecht, K. F. , R. Goelz , A. Bevot , I. Krageloh‐Mann , M. Wilke , and K. Lidzba . 2015. “Postnatal *Human Cytomegalovirus* Infection in Preterm Infants Has Long‐Term Neuropsychological Sequelae.” Journal of Pediatrics 166, no. 4: 834–839. e831. 25466679 10.1016/j.jpeds.2014.11.002

[brb370985-bib-0022] Britt, W. 2015. “Controversies in the Natural History of Congenital *Human Cytomegalovirus* Infection: The Paradox of Infection and Disease in Offspring of Women With Immunity Prior to Pregnancy.” Medical Microbiology and Immunology 204, no. 3: 263–271. 25764180 10.1007/s00430-015-0399-9

[brb370985-bib-0023] Brouwer, R. M. , A. M. Hedman , N. E. van Haren , et al. 2014. “Heritability of Brain Volume Change and Its Relation to Intelligence.” NeuroImage 100: 676–683. 24816534 10.1016/j.neuroimage.2014.04.072

[brb370985-bib-0024] Bryant, P. , C. Morley , S. Garland , and N. Curtis . 2002. “Cytomegalovirus Transmission From Breast Milk in Premature Babies: Does It Matter?” Archives of Disease in Childhood Fetal and Neonatal Edition 87, no. 2: F75–F77. 12193509 10.1136/fn.87.2.F75 PMC1721460

[brb370985-bib-0025] Buxmann, H. , M. Falk , R. Goelz , K. Hamprecht , C. F. Poets , and R. L. Schloesser . 2010. “Feeding of Very Low Birth Weight Infants Born to HCMV‐seropositive Mothers in Germany, Austria and Switzerland.” Acta Paediatrica 99, no. 12: 1819–1823. 20670309 10.1111/j.1651-2227.2010.01954.x

[brb370985-bib-0026] Chatzakis, C. , A. Sotiriadis , K. Dinas , and Y. Ville . 2023. “Neonatal and Long‐Term Outcomes of Infants With Congenital Cytomegalovirus Infection and Negative Amniocentesis: Systematic Review and Meta‐Analysis.” Ultrasound in Obstetrics and Gynecology: The Official Journal of the International Society of Ultrasound in Obstetrics and Gynecology 61, no. 2: 158–167. 36412976 10.1002/uog.26128 PMC10107880

[brb370985-bib-0027] Cheeran, M. C. , J. R. Lokensgard , and M. R. Schleiss . 2009. “Neuropathogenesis of Congenital Cytomegalovirus Infection: Disease Mechanisms and Prospects for Intervention.” Clinical Microbiology Reviews 22, no. 1: 99–126. Table of Contents. 19136436 10.1128/CMR.00023-08 PMC2620634

[brb370985-bib-0028] Craig, A. D. 2009. “How Do You Feel—now? The Anterior Insula and Human Awareness.” Nature Reviews Neuroscience 10, no. 1: 59–70. 19096369 10.1038/nrn2555

[brb370985-bib-0029] Dalton, M. A. , M. Hornberger , and O. Piguet . 2016. “Material Specific Lateralization of Medial Temporal Lobe Function: an fMRI Investigation.” Human Brain Mapping 37, no. 3: 933–941. 26700110 10.1002/hbm.23077 PMC6867313

[brb370985-bib-0030] Desimone, R. 1998. “Visual Attention Mediated by Biased Competition in Extrastriate Visual Cortex.” Philosophical Transactions of the Royal Society of London Series B, Biological Sciences 353, no. 1373: 1245–1255. 9770219 10.1098/rstb.1998.0280 PMC1692333

[brb370985-bib-0031] de Vries, J. J. , A. M. Korver , P. H. Verkerk , et al. 2011. “Congenital cytomegalovirus Infection in the Netherlands: Birth Prevalence and Risk Factors.” Journal of Medical Virology 83, no. 10: 1777–1782. 21837795 10.1002/jmv.22181

[brb370985-bib-0032] Dorn, M. , K. Lidzba , A. Bevot , R. Goelz , T. K. Hauser , and M. Wilke . 2014. “Long‐Term Neurobiological Consequences of Early Postnatal hCMV‐infection in Former Preterms: A Functional MRI Study.” Human Brain Mapping 35, no. 6: 2594–2606. 24027137 10.1002/hbm.22352 PMC6869207

[brb370985-bib-0033] Enders, G. , A. Daiminger , U. Bader , S. Exler , and M. Enders . 2011. “Intrauterine Transmission and Clinical Outcome of 248 Pregnancies With Primary Cytomegalovirus Infection in Relation to Gestational Age.” Journal of Clinical Virology: The Official Publication of the Pan American Society for Clinical Virology 52, no. 3: 244–246. 21820954 10.1016/j.jcv.2011.07.005

[brb370985-bib-0034] Fischer, C. , P. Meylan , and M. Bickle Graz , et al. 2012. “Severe Postnatally Acquired Cytomegalovirus Infection Presenting With Colitis, Pneumonitis and Sepsis‐Like Syndrome in an Extremely Low Birthweight Infant.” Neonatology 97: 339–345. 10.1159/000260137 19940517

[brb370985-bib-0035] Foulon, I. , A. Naessens , W. Foulon , A. Casteels , and F. Gordts . 2008. “Hearing Loss in Children With Congenital Cytomegalovirus Infection in Relation to the Maternal Trimester in Which the Maternal Primary Infection Occurred.” Pediatrics 122, no. 6: e1123–e1127. 19047212 10.1542/peds.2008-0770

[brb370985-bib-0036] Franke, K. , E. Luders , A. May , M. Wilke , and C. Gaser . 2012. “Brain Maturation: Predicting Individual BrainAGE in Children and Adolescents Using Structural MRI.” NeuroImage 63, no. 3: 1305–1312. 22902922 10.1016/j.neuroimage.2012.08.001

[brb370985-bib-0037] Frye, R. E. , B. Malmberg , P. Swank , K. Smith , and S. Landry . 2010. “Preterm Birth and Maternal Responsiveness During Childhood Are Associated With Brain Morphology in Adolescence.” Journal of the International Neuropsychological Society 16, no. 05: 784–794. 20609271 10.1017/S1355617710000585

[brb370985-bib-0038] Gabrielli, L. , M. P. Bonasoni , D. Santini , et al. 2012. “Congenital Cytomegalovirus Infection: Patterns of Fetal Brain Damage.” Clinical Microbiology and Infection: The Official Publication of the European Society of Clinical Microbiology and Infectious Diseases 18, no. 10: E419–427. 22882294 10.1111/j.1469-0691.2012.03983.x

[brb370985-bib-0039] Gaser, C. , R. Dahnke , P. M. Thompson , F. Kurth , and E. Luders . 2024. “The Alzheimer's Disease Neuroimaging Initiative CAT: a computational anatomy toolbox for the analysis of structural MRI data.” Gigascience. Jan 2; 13: giae049. 39102518 10.1093/gigascience/giae049 PMC11299546

[brb370985-bib-0040] Gaser, C. , and F. Kurth . 2016. "CAT 12 Manual." Accessed file:///C:/Util/spm12/toolbox/cat12/html/cat.html.

[brb370985-bib-0041] Gaser, C. 2018. "C. A. T." Accessed February 21, 2019. http://dbm.neuro.uni‐jena.de/cat/index.html.

[brb370985-bib-0042] Gentile, M. A. , T. J. Boll , S. Stagno , and R. F. Pass . 1989. “Intellectual Ability of Children After Perinatal cytomegalovirus Infection.” Developmental Medicine and Child Neurology 31, no. 6: 782–786. 2557252 10.1111/j.1469-8749.1989.tb04074.x

[brb370985-bib-0043] Giedd, J. N. , J. Blumenthal , N. O. Jeffries , et al. 1999. “Brain Development During Childhood and Adolescence: A Longitudinal MRI Study.” Nature Neuroscience 2, no. 10: 861–863. 10491603 10.1038/13158

[brb370985-bib-0044] Gimenez, M. , C. Junque , P. Vendrell , et al. 2006. “Abnormal Orbitofrontal Development due to Prematurity.” Neurology 67, no. 10: 1818–1822. 17130415 10.1212/01.wnl.0000244485.51898.93

[brb370985-bib-0045] Goelz, R. , C. Meisner , A. Bevot , K. Hamprecht , I. Kraegeloh‐Mann , and C. F. Poets . 2013. “Long‐Term Cognitive and Neurological Outcome of Preterm Infants With Postnatally Acquired CMV Infection Through Breast Milk.” Archives of Disease in Childhood Fetal and Neonatal Edition 98, no. 5: F430–F433. 23603882 10.1136/archdischild-2012-303384

[brb370985-bib-0046] Gousias, I. S. , A. D. Edwards , M. A. Rutherford , et al. 2012. “Magnetic Resonance Imaging of the Newborn Brain: Manual Segmentation of Labelled Atlases in Term‐born and Preterm Infants.” NeuroImage 62, no. 3: 1499–1509. 22713673 10.1016/j.neuroimage.2012.05.083

[brb370985-bib-0047] Groeschel, S. , B. Vollmer , M. D. King , and A. Connelly . 2010. “Developmental Changes in Cerebral Grey and White Matter Volume From Infancy to Adulthood.” International Journal of Developmental Neuroscience 28, no. 6: 481–489. 20600789 10.1016/j.ijdevneu.2010.06.004

[brb370985-bib-0048] Hamele, M. , R. Flanagan , C. A. Loomis , T. Stevens , and M. P. Fairchok . 2010. “Severe Morbidity and Mortality With Breast Milk Associated Cytomegalovirus Infection.” The Pediatric Infectious Disease Journal 29, no. 1: 84–86. 19884873 10.1097/INF.0b013e3181b6dbb5

[brb370985-bib-0049] Hamprecht, K. , and R. Goelz . 2017. “Postnatal Cytomegalovirus Infection Through Human Milk in Preterm Infants: Transmission, Clinical Presentation, and Prevention.” Clinics in Perinatology 44, no. 1: 121–130. 28159200 10.1016/j.clp.2016.11.012

[brb370985-bib-0050] Hamprecht, K. , J. Maschmann , M. Vochem , K. Dietz , C. P. Speer , and G. Jahn . 2001. “Epidemiology of Transmission of Cytomegalovirus From Mother to Preterm Infant by Breastfeeding.” Lancet 357, no. 9255: 513–518. 11229670 10.1016/S0140-6736(00)04043-5

[brb370985-bib-0051] Harrison, M. S. , and R. L. Goldenberg . 2016. “Global Burden of Prematurity.” Seminars in Fetal and Neonatal Medicine 21, no. 2: 74–79. 26740166 10.1016/j.siny.2015.12.007

[brb370985-bib-0052] Helenius, P. , P. Sivonen , T. Parviainen , et al. 2014. “Abnormal Functioning of the Left Temporal Lobe in Language‐impaired Children.” Brain and Language 130: 11–18. 24568877 10.1016/j.bandl.2014.01.005

[brb370985-bib-0053] Hutchinson, E. A. , C. R. De Luca , L. W. Doyle , G. Roberts , and P. J. Anderson . 2013. “School‐age Outcomes of Extremely Preterm or Extremely Low Birth Weight Children.” Pediatrics 131, no. 4: e1053–1061. 23509167 10.1542/peds.2012-2311

[brb370985-bib-0054] Inder, T. E. , J. J. Volpe , and P. J. Anderson . 2023. “Defining the Neurologic Consequences of Preterm Birth.” New England Journal of Medicine 389, no. 5: 441–453. 37530825 10.1056/NEJMra2303347

[brb370985-bib-0055] Jacobson, L. , B. Vollmer , A. Kistner , and B. Böhm . 2021. “Severity of Retinopathy of Prematurity Was Associated With a Higher Risk of Cerebral Dysfunction in Young Adults Born Extremely Preterm.” Acta Paediatrica 110, no. 2: 528–536. 32628800 10.1111/apa.15461

[brb370985-bib-0056] Jeneson, A. , and L. R. Squire . 2012. “Working Memory, Long‐Term Memory, and Medial Temporal Lobe Function.” Learning and Memory 19, no. 1: 15–25. 22180053 10.1101/lm.024018.111 PMC3246590

[brb370985-bib-0057] Jim, W. T. , N. C. Chiu , C. S. Ho , et al. 2015. “Outcome of Preterm Infants With Postnatal Cytomegalovirus Infection via Breast Milk: A Two‐Year Prospective Follow‐Up Study.” Medicine 94, no. 43: e1835. 26512588 10.1097/MD.0000000000001835 PMC4985402

[brb370985-bib-0058] Jim, W. T. , C. H. Shu , N. C. Chiu , et al. 2009. “High Cytomegalovirus Load and Prolonged Virus Excretion in Breast Milk Increase Risk for Viral Acquisition by Very Low Birth Weight Infants.” The Pediatric Infectious Disease Journal 28, no. 10: 891–894. 19687768 10.1097/INF.0b013e3181a55c52

[brb370985-bib-0059] Joel, D. , Z. Berman , I. Tavor , et al. 2015. “Sex Beyond the Genitalia: The Human Brain Mosaic.” PNAS 112, no. 50: 15468–15473. 26621705 10.1073/pnas.1509654112 PMC4687544

[brb370985-bib-0060] Johnson, S. , and N. Marlow . 2017. “Early and Long‐Term Outcome of Infants Born Extremely Preterm.” Archives of Disease in Childhood 102, no. 1: 97–102. 27512082 10.1136/archdischild-2015-309581

[brb370985-bib-0061] Johnson, S. J. , H. Hosford‐Dunn , S. Paryani , A. Yeager , and N. Malachowski . 1986. “Prevalence of Sensorineural Hearing Loss in Premature and Sick Term Infants With Perinatally Acquired Cytomegalovirus Infection.” Ear and Hearing 7, no. 5: 325–327. 3021553 10.1097/00003446-198610000-00007

[brb370985-bib-0062] Josephson, C. D. , A. M. Caliendo , K. A. Easley , et al. 2014. “Blood Transfusion and Breast Milk Transmission of Cytomegalovirus in Very Low‐birth‐weight Infants: A Prospective Cohort Study.” JAMA Pediatrics 168, no. 11: 1054–1062. 25243446 10.1001/jamapediatrics.2014.1360 PMC4392178

[brb370985-bib-0063] Kenneson, A. , and M. J. Cannon . 2007. “Review and Meta‐Analysis of the Epidemiology of Congenital Cytomegalovirus (CMV) Infection.” Reviews in Medical Virology 17, no. 4: 253–276. 17579921 10.1002/rmv.535

[brb370985-bib-0064] Kesler, S. R. , A. L. Reiss , B. Vohr , et al. 2008. “Brain Volume Reductions Within Multiple Cognitive Systems in Male Preterm Children at Age Twelve.” Journal of Pediatrics 152, no. 4: 513–520. 18346506 10.1016/j.jpeds.2007.08.009 PMC3270939

[brb370985-bib-0065] Klotz, D. , S. Jansen , C. Gebauer , and H. Fuchs . 2018. “Handling of Breast Milk by Neonatal Units: Large Differences in Current Practices and Beliefs.” Frontiers in Pediatrics 6: 235. 30234076 10.3389/fped.2018.00235 PMC6131667

[brb370985-bib-0066] Kostovic, I. , and M. Judas . 2002. “Correlation Between the Sequential Ingrowth of Afferents and Transient Patterns of Cortical Lamination in Preterm Infants.” The Anatomical Record 267, no. 1: 1–6. 11984786 10.1002/ar.10069

[brb370985-bib-0067] Kumar, M. L. , G. A. Nankervis , I. B. Jacobs , et al. 1984. “Congenital and Postnatally Acquired cytomegalovirus Infections: Long‐Term Follow‐up.” Journal of Pediatrics 104, no. 5: 674–679. 6325654 10.1016/s0022-3476(84)80942-7

[brb370985-bib-0068] Kurath, S. , G. Halwachs‐Baumann , W. Muller , and B. Resch . 2010. “Transmission of Cytomegalovirus via Breast Milk to the Prematurely Born Infant: A Systematic Review.” Clinical Microbiology and Infection: The Official Publication of the European Society of Clinical Microbiology and Infectious Diseases 16, no. 8: 1172–1178. 20670291 10.1111/j.1469-0691.2010.03140.x

[brb370985-bib-0069] Lanzieri, T. M. , S. C. Dollard , S. R. Bialek , and S. D. Grosse . 2014. “Systematic Review of the Birth Prevalence of Congenital cytomegalovirus Infection in Developing Countries.” International Journal of Infectious Diseases (IJID): Official Publication of the International Society for Infectious Diseases 22: 44–48. 24631522 10.1016/j.ijid.2013.12.010 PMC4829484

[brb370985-bib-0070] Lanzieri, T. M. , S. C. Dollard , C. D. Josephson , D. S. Schmid , and S. R. Bialek . 2013. “Breast Milk‐acquired Cytomegalovirus Infection and Disease in VLBW and Premature Infants.” Pediatrics 131, no. 6: e1937–e1945. 23713111 10.1542/peds.2013-0076 PMC4850548

[brb370985-bib-0071] Leviton, A. , R. N. Fichorova , and T. M. O'Shea , et al. 2013. “Two‐hit Model of Brain Damage in the Very Preterm Newborn: Small for Gestational Age and Postnatal Systemic Inflammation.” Pediatric Research 73, no. 3: 362–370. 23364171 10.1038/pr.2012.188 PMC3642985

[brb370985-bib-0072] Li, H. , L. D. Nickerson , T. E. Nichols , and J. H. Gao . 2017. “Comparison of a Non‐stationary Voxelation‐corrected Cluster‐size Test With TFCE for Group‐Level MRI Inference.” Human Brain Mapping 38, no. 3: 1269–1280. 27785843 10.1002/hbm.23453 PMC6866890

[brb370985-bib-0073] Luders, E. , P. M. Thompson , K. L. Narr , A. W. Toga , L. Jancke , and C. Gaser . 2006. “A Curvature‐based Approach to Estimate Local Gyrification on the Cortical Surface.” NeuroImage 29, no. 4: 1224–1230. 16223589 10.1016/j.neuroimage.2005.08.049

[brb370985-bib-0074] Malone, I. B. , K. K. Leung , S. Clegg , et al. 2015. “Accurate Automatic Estimation of Total Intracranial Volume: A Nuisance Variable With Less Nuisance.” NeuroImage 104: 366–372. 25255942 10.1016/j.neuroimage.2014.09.034 PMC4265726

[brb370985-bib-0075] Marslen‐Wilson, W. , and L. K. Tyler . 1980. “The Temporal Structure of Spoken Language Understanding.” Cognition 8, no. 1: 1–71. 7363578 10.1016/0010-0277(80)90015-3

[brb370985-bib-0076] Martinussen, M. , B. Fischl , H. B. Larsson , et al. 2005. “Cerebral Cortex Thickness in 15‐year‐old Adolescents With Low Birth Weight Measured by an Automated MRI‐based Method.” Brain 128, no. 11: 2588–2596. 16123146 10.1093/brain/awh610

[brb370985-bib-0077] Maschmann, J. , D. Müller , K. Lazar , R. Goelz , and K. Hamprecht . 2019. “New Short‐Term Heat Inactivation Method of Cytomegalovirus (CMV) in Breast Milk: Impact on CMV Inactivation, CMV Antibodies and Enzyme Activities.” Archives of Disease in Childhood Fetal and Neonatal Edition 104, no. 6: F604–F608. 30728181 10.1136/archdischild-2018-316117

[brb370985-bib-0078] Meng, Y. , G. Li , I. Rekik , et al. 2017. “Can We Predict Subject‐specific Dynamic Cortical Thickness Maps During Infancy From Birth?” Human Brain Mapping 38, no. 6: 2865–2874. 28295833 10.1002/hbm.23555 PMC5426957

[brb370985-bib-0079] Menon, V. , and L. Q. Uddin . 2010. “Saliency, Switching, Attention and Control: A Network Model of Insula Function.” Brain Structure and Function 214, no. 5‐6: 655–667. 20512370 10.1007/s00429-010-0262-0 PMC2899886

[brb370985-bib-0080] Miron, D. , S. Brosilow , K. Felszer , et al. 2005. “Incidence and Clinical Manifestations of Breast Milk‐acquired Cytomegalovirus Infection in Low Birth Weight Infants.” Journal of Perinatology: Official Journal of the California Perinatal Association 25, no. 5: 299–303. 15674408 10.1038/sj.jp.7211255

[brb370985-bib-0081] Monson, B. B. , P. J. Anderson , L. G. Matthews , et al. 2016. “Examination of the Pattern of Growth of Cerebral Tissue Volumes from Hospital Discharge to Early Childhood in Very Preterm Infants.” JAMA Pediatrics 170, no. 8: 772–779. 27368090 10.1001/jamapediatrics.2016.0781

[brb370985-bib-0082] Monzalvo, K. , and G. Dehaene‐Lambertz . 2013. “How Reading Acquisition Changes Children's Spoken Language Network.” Brain and Language 127, no. 3: 356–365. 24216407 10.1016/j.bandl.2013.10.009

[brb370985-bib-0083] Moster, D. , R. T. Lie , and T. Markestad . 2008. “Long‐Term Medical and Social Consequences of Preterm Birth.” New England Journal of Medicine 359, no. 3: 262–273. 18635431 10.1056/NEJMoa0706475

[brb370985-bib-0084] Murner‐Lavanchy, I. , M. Steinlin , M. Nelle , et al. 2014. “Delay of Cortical Thinning in Very Preterm Born Children.” Early Human Development 90, no. 9: 443–450. 24976634 10.1016/j.earlhumdev.2014.05.013

[brb370985-bib-0085] Nagy, Z. , H. Lagercrantz , and C. Hutton . 2011. “Effects of Preterm Birth on Cortical Thickness Measured in Adolescence.” Cerebral Cortex 21, no. 2: 300–306. 20522538 10.1093/cercor/bhq095 PMC3020580

[brb370985-bib-0086] Naing, Z. W. , G. M. Scott , A. Shand , et al. 2016. “Congenital Cytomegalovirus Infection in Pregnancy: A Review of Prevalence, Clinical Features, Diagnosis and Prevention.” The Australian and New Zealand Journal of Obstetrics and Gynaecology 56, no. 1: 9–18. 26391432 10.1111/ajo.12408

[brb370985-bib-0087] Nam, K. W. , N. Castellanos , A. Simmons , et al. 2015. “Alterations in Cortical Thickness Development in Preterm‐born Individuals: Implications for High‐order Cognitive Functions.” NeuroImage 115: 64–75. 25871628 10.1016/j.neuroimage.2015.04.015 PMC4463853

[brb370985-bib-0088] Neuberger, P. , K. Hamprecht , M. Vochem , et al. 2006. “Case‐control Study of Symptoms and Neonatal Outcome of Human Milk‐transmitted Cytomegalovirus Infection in Premature Infants.” Journal of Pediatrics 148, no. 3: 326–331. 16615961 10.1016/j.jpeds.2005.09.030

[brb370985-bib-0089] Nichols, T. , and S. Hayasaka . 2003. “Controlling the Familywise Error Rate in Functional Neuroimaging: A Comparative Review.” Statistical Methods in Medical Research 12, no. 5: 419–446. 14599004 10.1191/0962280203sm341ra

[brb370985-bib-0090] Nijman, J. , L. S. de Vries , C. Koopman‐Esseboom , C. S. Uiterwaal , A. M. van Loon , and M. A. Verboon‐Maciolek . 2012. “Postnatally Acquired Cytomegalovirus Infection in Preterm Infants: A Prospective Study on Risk Factors and Cranial Ultrasound Findings.” Archives of Disease in Childhood Fetal and Neonatal Edition 97, no. 4: F259–263. 22247412 10.1136/archdischild-2011-300405

[brb370985-bib-0091] Nijman, J. , J. Gunkel , L. S. de Vries , et al. 2013. “Reduced Occipital Fractional Anisotropy on Cerebral Diffusion Tensor Imaging in Preterm Infants With Postnatally Acquired Cytomegalovirus Infection.” Neonatology 104, no. 2: 143–150. 23887677 10.1159/000351017

[brb370985-bib-0092] Nosarti, C. , E. Giouroukou , E. Healy , et al. 2008. “Grey and White Matter Distribution in Very Preterm Adolescents Mediates Neurodevelopmental Outcome.” Brain 131, no. 1: 205–217. 18056158 10.1093/brain/awm282

[brb370985-bib-0093] Nosarti, C. , K. W. Nam , M. Walshe , et al. 2014. “Preterm Birth and Structural Brain Alterations in Early Adulthood.” NeuroImage: Clinical 6: 180–191. 25379430 10.1016/j.nicl.2014.08.005 PMC4215396

[brb370985-bib-0094] Oldfield, R. C. 1971. “The Assessment and Analysis of Handedness: The Edinburgh Inventory.” Neuropsychologia 9, no. 1: 97–113. 5146491 10.1016/0028-3932(71)90067-4

[brb370985-bib-0095] Oosterom, N. , J. Nijman , J. Gunkel , et al. 2015. “Neuro‐imaging Findings in Infants With Congenital Cytomegalovirus Infection: Relation to Trimester of Infection.” Neonatology 107, no. 4: 289–296. 25790782 10.1159/000375439

[brb370985-bib-0096] Padilla, N. , G. Alexandrou , M. Blennow , H. Lagercrantz , and U. Aden . 2015. “Brain Growth Gains and Losses in Extremely Preterm Infants at Term.” Cerebral Cortex 25, no. 7: 1897–1905. 24488941 10.1093/cercor/bht431

[brb370985-bib-0097] Paryani, S. G. , A. S. Yeager , H. Hosford‐Dunn , et al. 1985. “Sequelae of Acquired Cytomegalovirus Infection in Premature and Sick Term Infants.” Journal of Pediatrics 107, no. 3: 451–456. 2993576 10.1016/s0022-3476(85)80533-3

[brb370985-bib-0098] Patra, K. , M. M. Greene , A. L. Patel , and P. Meier . 2016. “Maternal Education Level Predicts Cognitive, Language, and Motor Outcome in Preterm Infants in the Second Year of Life.” American Journal of Perinatology 33, no. 8: 738–744. 26890439 10.1055/s-0036-1572532 PMC4919155

[brb370985-bib-0099] Pavlova, M. , A. Sokolov , and I. Krageloh‐Mann . 2007. “Visual Navigation in Adolescents With Early Periventricular Lesions: Knowing Where, but Not Getting There.” Cerebral Cortex 17, no. 2: 363–369. 16525128 10.1093/cercor/bhj153

[brb370985-bib-0100] Pavlova, M. , M. Staudt , A. Sokolov , N. Birbaumer , and I. Krageloh‐Mann . 2003. “Perception and Production of Biological Movement in Patients With Early Periventricular Brain Lesions.” Brain 126, no. 3: 692–701. 12566289 10.1093/brain/awg062

[brb370985-bib-0101] Pellkofer, Y. , M. Hammerl , E. Griesmaier , et al. 2023. “The Effect of Postnatal Cytomegalovirus Infection on (Micro)Structural Cerebral Development in Very Preterm Infants at Term‐Equivalent Age.” Neonatology 120, no. 6: 727–735. 37634498 10.1159/000532084

[brb370985-bib-0102] Pernet, C. R. 2016. “The General Lineal Model: Theory and Practicalities in Brain Morphometric Analysis.” In Brain Morphometry: Methods and Clinical Applications, G. Spalletta , T. Gili , and F. Piras , Springer.

[brb370985-bib-0103] Petermann, F. , and U. Petermann . 2008. “HAWIK‐IV.” Kindheit Und Entwicklung 17, no. 2: 71–75.

[brb370985-bib-0104] Peters, M. , L. Jancke , J. F. Staiger , G. Schlaug , Y. Huang , and H. Steinmetz . 1998. “Unsolved Problems in Comparing Brain Sizes in Homo Sapiens.” Brain and Cognition 37, no. 2: 254–285. 9665746 10.1006/brcg.1998.0983

[brb370985-bib-0105] Raju, T. N. , V. L. Pemberton , S. Saigal , C. J. Blaisdell , M. Moxey‐Mims , and S. Buist . 2017. “Long‐Term Healthcare Outcomes of Preterm Birth: An Executive Summary of a Conference Sponsored by the National Institutes of Health.” Journal of Pediatrics 181: 309–318. e301. 27806833 10.1016/j.jpeds.2016.10.015

[brb370985-bib-0106] Rakic, P. 1995. “A Small Step for the Cell, a Giant Leap for Mankind: A Hypothesis of Neocortical Expansion During Evolution.” Trends in Neurosciences 18, no. 9: 383–388. 7482803 10.1016/0166-2236(95)93934-p

[brb370985-bib-0107] Raznahan, A. , P. Shaw , F. Lalonde , et al. 2011. “How Does Your Cortex Grow?” The Journal of Neuroscience: The Official Journal of the Society for Neuroscience 31, no. 19: 7174–7177. 21562281 10.1523/JNEUROSCI.0054-11.2011 PMC3157294

[brb370985-bib-0108] Rogers, R. D. , A. M. Owen , H. C. Middleton , et al. 1999. “Choosing Between Small, Likely Rewards and Large, Unlikely Rewards Activates Inferior and Orbital Prefrontal Cortex.” The Journal of Neuroscience: The Official Journal of the Society for Neuroscience 19, no. 20: 9029–9038. 10516320 10.1523/JNEUROSCI.19-20-09029.1999 PMC6782753

[brb370985-bib-0109] Ruiz, M. , P. Goldblatt , J. Morrison , et al. 2015. “Mother's Education and the Risk of Preterm and Small for Gestational Age Birth: A DRIVERS Meta‐Analysis of 12 European Cohorts.” Journal of Epidemiology and Community Health 69, no. 9: 826–833. 25911693 10.1136/jech-2014-205387 PMC4552914

[brb370985-bib-0110] Salimi‐Khorshidi, G. , S. M. Smith , and T. E. Nichols . 2011. “Adjusting the Effect of Nonstationarity in Cluster‐based and TFCE Inference.” NeuroImage 54, no. 3: 2006–2019. 20955803 10.1016/j.neuroimage.2010.09.088

[brb370985-bib-0111] Scheuchenegger, A. , E. Lechner , G. Wiesinger‐Eidenberger , et al. 2014. “Short‐Term Morbidities in Moderate and Late Preterm Infants.” Klinische Padiatrie 226, no. 4: 216–220. 24158889 10.1055/s-0033-1355394

[brb370985-bib-0112] Schill, S. , T. Thomas , D. Münch , and G. Heller . 2017. Qualitätsreport 2016. Gemeinsamer Bundesausschuss. available online at https://iqtig.org/downloads/berichte/2016/IQTIG_Qualitaetsreport-2016.pdf.

[brb370985-bib-0113] Schleiss, M. R. 2006. “Acquisition of *Human Cytomegalovirus* Infection in Infants via Breast Milk: Natural Immunization or Cause for Concern?” Reviews in Medical Virology 16, no. 2: 73–82. 16287195 10.1002/rmv.484

[brb370985-bib-0114] Schnack, H. G. , N. E. van Haren , R. M. Brouwer , et al. 2015. “Changes in Thickness and Surface Area of the Human Cortex and Their Relationship With Intelligence.” Cerebral Cortex 25, no. 6: 1608–1617. 24408955 10.1093/cercor/bht357

[brb370985-bib-0115] Scott, M. , D. Flaherty , and J. Currall . 2014. “Statistics: General Linear Models (a flexible approach).” The Journal of Small Animal Practice 55, no. 10: 527–530. 25134691 10.1111/jsap.12260

[brb370985-bib-0116] Shaw, P. , D. Greenstein , J. Lerch , et al. 2006. “Intellectual Ability and Cortical Development in Children and Adolescents.” Nature 440, no. 7084: 676–679. 16572172 10.1038/nature04513

[brb370985-bib-0117] Simister, N. E. 2003. “Placental Transport of Immunoglobulin G.” Vaccine 21: 3365–3369. 12850341 10.1016/s0264-410x(03)00334-7

[brb370985-bib-0118] Smith, S. M. , and T. E. Nichols . 2009. “Threshold‐free Cluster Enhancement: Addressing Problems of Smoothing, Threshold Dependence and Localisation in Cluster Inference.” NeuroImage 44, no. 1: 83–98. 18501637 10.1016/j.neuroimage.2008.03.061

[brb370985-bib-0119] Soria‐Pastor, S. , N. Padilla , L. Zubiaurre‐Elorza , et al. 2009. “Decreased Regional Brain Volume and Cognitive Impairment in Preterm Children at Low Risk.” Pediatrics 124, no. 6: e1161–e1170. 19948618 10.1542/peds.2009-0244

[brb370985-bib-0120] Stagno, S. , D. M. Brasfield , M. B. Brown , et al. 1981. “Infant Pneumonitis Associated With Cytomegalovirus, Chlamydia, Pneumocystis, and Ureaplasma: A Prospective Study.” Pediatrics 68, no. 3: 322–329. 6269042

[brb370985-bib-0121] Stagno, S. , and G. A. Cloud . 1994. “Working Parents: the Impact of Day Care and Breast‐feeding on cytomegalovirus Infections in Offspring.” PNAS 91, no. 7: 2384–2389. 8146127 10.1073/pnas.91.7.2384 PMC43376

[brb370985-bib-0122] Stoll, B. J. , N. I. Hansen , E. F. Bell , et al. 2010. “Neonatal Outcomes of Extremely Preterm Infants From the NICHD Neonatal Research Network.” Pediatrics 126, no. 3: 443–456. 20732945 10.1542/peds.2009-2959 PMC2982806

[brb370985-bib-0123] Takahashi, R. , M. Tagawa , M. Sanjo , et al. 2007. “Severe Postnatal cytomegalovirus Infection in a Very Premature Infant.” Neonatology 92, no. 4: 236–239. 17570943 10.1159/000103982

[brb370985-bib-0124] Teissier, N. , C. Fallet‐Bianco , A. L. Delezoide , et al. 2014. “Cytomegalovirus‐induced Brain Malformations in Fetuses.” Journal of Neuropathology and Experimental Neurology 73, no. 2: 143–158. 24423639 10.1097/NEN.0000000000000038

[brb370985-bib-0125] Thompson, D. K. , S. K. Warfield , J. B. Carlin , et al. 2007. “Perinatal Risk Factors Altering Regional Brain Structure in the Preterm Infant.” Brain 130, no. 3: 667–677. 17008333 10.1093/brain/awl277

[brb370985-bib-0126] Townsend, C. L. , M. Forsgren , K. Ahlfors , S. A. Ivarsson , P. A. Tookey , and C. S. Peckham . 2013. “Long‐term Outcomes of Congenital Cytomegalovirus Infection in Sweden and the United Kingdom.” Clinical Infectious Diseases: An Official Publication of the Infectious Diseases Society of America 56, no. 9: 1232–1239. 23334811 10.1093/cid/cit018 PMC3616516

[brb370985-bib-0127] van der Strate, B. W. , M. C. Harmsen , P. Schafer , et al. 2001. “Viral Load in Breast Milk Correlates With Transmission of *Human Cytomegalovirus* to Preterm Neonates, but Lactoferrin Concentrations Do Not.” Clinical and Diagnostic Laboratory Immunology 8, no. 4: 818–821. 11427433 10.1128/CDLI.8.4.818-821.2001 PMC96149

[brb370985-bib-0128] van der Voorn, J. P. , P. J. Pouwels , R. J. Vermeulen , F. Barkhof , and M. S. van der Knaap . 2009. “Quantitative MR Imaging and Spectroscopy in Congenital Cytomegalovirus Infection and Periventricular Leukomalacia Suggests a Comparable Neuropathological Substrate of the Cerebral White Matter Lesions.” Neuropediatrics 40, no. 4: 168–173. 20135574 10.1055/s-0029-1243228

[brb370985-bib-0129] Vijayakumar, N. , N. B. Allen , G. Youssef , et al. 2016. “Brain Development During Adolescence: A Mixed‐Longitudinal Investigation of Cortical Thickness, Surface Area, and Volume.” Human Brain Mapping 37, no. 6: 2027–2038. 26946457 10.1002/hbm.23154 PMC6867680

[brb370985-bib-0130] Vollmer, B. , K. Seibold‐Weiger , C. Schmitz‐Salue , et al. 2004. “Postnatally Acquired Cytomegalovirus Infection via Breast Milk: Effects on Hearing and Development in Preterm Infants.” The Pediatric Infectious Disease Journal 23, no. 4: 322–327. 15071286 10.1097/00006454-200404000-00009

[brb370985-bib-0131] Volpe, J. J. 2009. “Brain Injury in Premature Infants: A Complex Amalgam of Destructive and Developmental Disturbances.” The Lancet Neurology 8, no. 1: 110–124. 19081519 10.1016/S1474-4422(08)70294-1 PMC2707149

[brb370985-bib-0132] Wang, C. , X. Zhang , S. Bialek , and M. J. Cannon . 2011. “Attribution of Congenital Cytomegalovirus Infection to Primary versus Non‐Primary Maternal Infection.” Clinical Infectious Diseases: An Official Publication of the Infectious Diseases Society of America 52, no. 2: e11–e13. 21288834 10.1093/cid/ciq085

[brb370985-bib-0133] Wierenga, L. M. , M. Langen , B. Oranje , and S. Durston . 2014. “Unique Developmental Trajectories of Cortical Thickness and Surface Area.” NeuroImage 87: 120–126. 24246495 10.1016/j.neuroimage.2013.11.010

[brb370985-bib-0134] Wilke, M. , T. K. Hauser , I. Krageloh‐Mann , and K. Lidzba . 2014. “Specific Impairment of Functional Connectivity Between Language Regions in Former Early Preterms.” Human Brain Mapping 35, no. 7: 3372–3384. 24243552 10.1002/hbm.22408 PMC6869459

[brb370985-bib-0138] Wilke, M. , S. K. Holland , M. Altaye , and C. Gaser . 2008. “Template-O-Matic: A toolbox for creating customized pediatric templates.” NeuroImage 41, no. 3: 903–913. 18424084 10.1016/j.neuroimage.2008.02.056

[brb370985-bib-0135] Wilke, M. , I. Krageloh‐Mann , and S. K. Holland . 2007. “Global and Local Development of Gray and White Matter Volume in Normal Children and Adolescents.” Experimental Brain Research 178, no. 3: 296–307. 17051378 10.1007/s00221-006-0732-z PMC2265798

[brb370985-bib-0136] Yeager, A. S. , P. E. Palumbo , N. Malachowski , R. L. Ariagno , and D. K. Stevenson . 1983. “Sequelae of Maternally Derived Cytomegalovirus Infections in Premature Infants.” Journal of Pediatrics 102, no. 6: 918–922. 6304275 10.1016/s0022-3476(83)80025-0

[brb370985-bib-0137] Zubiaurre‐Elorza, L. , S. Soria‐Pastor , C. Junque , et al. 2011. “Gray Matter Volume Decrements in Preterm Children With Periventricular Leukomalacia.” Pediatric Research 69, no. 6: 554–560. 21386751 10.1203/PDR.0b013e3182182366

